# Central pathways causing fatigue in neuro-inflammatory and autoimmune illnesses

**DOI:** 10.1186/s12916-014-0259-2

**Published:** 2015-02-06

**Authors:** Gerwyn Morris, Michael Berk, Ken Walder, Michael Maes

**Affiliations:** 1 Tir Na Nog, Bryn Road seaside 87, Llanelli, SA152LW Wales UK; 2 IMPACT Strategic Research Centre, School of Medicine, Deakin University, Geelong, Australia; 3 Department of Psychiatry and The Florey Institute of Neuroscience and Mental Health, Orygen, The National Centre of Excellence in Youth Mental Health, The University of Melbourne, Parkville, Australia; 4 Centre for Molecular and Medical Research, School of Medicine, Deakin University, Geelong, Australia; 5 Department of Psychiatry, Faculty of Medicine, Chulalongkorn University, Bangkok, Thailand

**Keywords:** Immune, Inflammation, Oxidative stress, Toll-like receptor, Fatigue, Mitochondria, Multiple sclerosis, Chronic fatigue syndrome, Parkinson’s disease

## Abstract

**Background:**

The genesis of severe fatigue and disability in people following acute pathogen invasion involves the activation of Toll-like receptors followed by the upregulation of proinflammatory cytokines and the activation of microglia and astrocytes. Many patients suffering from neuroinflammatory and autoimmune diseases, such as multiple sclerosis, Parkinson’s disease and systemic lupus erythematosus, also commonly suffer from severe disabling fatigue. Such patients also present with chronic peripheral immune activation and systemic inflammation in the guise of elevated proinflammtory cytokines, oxidative stress and activated Toll-like receptors. This is also true of many patients presenting with severe, apparently idiopathic, fatigue accompanied by profound levels of physical and cognitive disability often afforded the non-specific diagnosis of chronic fatigue syndrome.

**Discussion:**

Multiple lines of evidence demonstrate a positive association between the degree of peripheral immune activation, inflammation and oxidative stress, gray matter atrophy, glucose hypometabolism and cerebral hypoperfusion in illness, such as multiple sclerosis, Parkinson’s disease and chronic fatigue syndrome. Most, if not all, of these abnormalities can be explained by a reduction in the numbers and function of astrocytes secondary to peripheral immune activation and inflammation. This is also true of the widespread mitochondrial dysfunction seen in otherwise normal tissue in neuroinflammatory, neurodegenerative and autoimmune diseases and in many patients with disabling, apparently idiopathic, fatigue. Given the strong association between peripheral immune activation and neuroinflammation with the genesis of fatigue the latter group of patients should be examined using FLAIR magnetic resonance imaging (MRI) and tested for the presence of peripheral immune activation.

**Summary:**

It is concluded that peripheral inflammation and immune activation, together with the subsequent activation of glial cells and mitochondrial damage, likely account for the severe levels of intractable fatigue and disability seen in many patients with neuroimmune and autoimmune diseases.This would also appear to be the case for many patients afforded a diagnosis of Chronic Fatigue Syndrome.

## Background

There is copious evidence establishing the causative role of peripheral immune activation and inflammation, evidenced by elevated levels of proinflammatory cytokines in the genesis of debilitating fatigue in neuro-inflammatory, autoimmune and inflammatory disorders [1,2]. Activation of pathogen recognition receptors by pathogen associated molecular patterns leads to the production of nuclear factor NF-kappaB and subsequent production of proinflammatory cytokines by the myeloid differentiation primary response gene (88) (MYD88), which is a universal adapter protein that is used by almost all Toll-like receptors (TLRs) in dependent and independent pathways [3-5]. Systemic inflammatory stimuli, resulting from the presence of proinflammatory cytokines in the peripheral circulation, enter the brain via a number of routes [1,6] activating microglia and astrocytes inducing the production of proinflammatory cytokines and other neurotoxins leading to an environment of neuroinflammation [7,8]. This sequence of events ultimately underpins the genesis of fatigue and other signs and symptoms associated with acute pathogen invasion [1,9,10]. Many people suffering from a range of neuroimmune and autoimmune diseases also suffer from debilitating or intractable fatigue.

The existence of chronically activated immune and inflammatory pathways in the periphery and their causative role in the genesis of neuroinflammation has been established in a range of neuroinflammatory and neurodegenerative diseases, such as multiple sclerosis, Alzheimer’s and Parkinson’s disease [11-16]. Many individuals with neuroinflammatory and neurodegenerative diseases also suffer from fatigue. For example, upwards of 80% of multiple sclerosis patients suffer from fatigue [17]. A study by Beiske and Svensson reported that between 37% and 57% of patients with Parkinson’s disease also experience incapacitating fatigue [18]. Fatigue is one of the characteristics of major depression [19,20]. Chronic systemic inflammation and the presence of activated microglia are also found in patients with major depression [19-22]. Chronic systemic inflammation and immune activation is also an invariant finding in many patients diagnosed with chronic fatigue syndrome (CFS) even without evidence of increased pathogen load [17].

Severe chronic fatigue is also experienced by many people with an autoimmune disease. Thus, upwards of 67% of people with Sjogren's syndrome [23], 76% of patients with systemic lupus erythromatosis (SLE) [24] and 70% of people with rheumatoid arthritis [25] suffer incapacitating levels of fatigue. Peripheral systemic inflammation and immune activation, as evidenced by elevated levels of proinflammatory cytokines and other inflammogens, is seen in patients with rheumatoid arthritis [26,27], SLE [28,29] and Sjogren's syndrome [30,31]. It is interesting to note that neurological sequelae are seen in up to 80% of patients with SLE and 70% of patients with primary Sjögren's syndrome [32,33]. In addition, the presence of neuroinflammation, in the shape of activated microglia, has been confirmed in patients with SLE [34]. Neurological complications are also commonplace in patients with rheumatoid arthritis [35].

The question arises as to the factors involved in creating a chronically activated immune system in these patients. While there is some evidence linking viral infections to the development of multiple sclerosis [36,37], the situation in Parkinson’s disease is different, where there is considerable evidence suggesting environmental toxins in the etiopathogenesis of the illness [38]. One of the key drivers in the development of chronic immune activation in the absence of bacteria or virus infection is the development of chronic inflammation as evidenced by elevated levels of cytokines and oxidative and nitrosative stress (O and NS) and characterized by activated NF-kappaB [6,39]. Indeed, the production of proinflammatory cytokines and other inflammatory molecules by macrophages and other sentinel cells, even in the absence of pathogen invasion, and the subsequent activation of NF-kappaB are early events in the genesis of chronic inflammation [40,41]. Activation of this transcription factor leads to the upregulation of cytokines and O and NS [6,42-44]. These players can engage in a feed-forward manner to maintain and amplify chronic inflammation and immune activation in a TLR radical cycle [4].

Briefly, elevated levels of proinflammatory cytokines can amplify the activity of NF-kappaB by stimulating the canonical pathway leading to a cycle of mutually elevated activity [45,46]. The relation between O and NS and NF-kappaB is a little more complex, but the upregulation of O and NS can directly increase the activity of NF-kappaB [47]. Moreover, O and NS may damage lipids, proteins and DNA, leading to the formation of redox-derived damage-associated molecular pattern molecules (DAMPs) [48,49]. Once formed, these redox-derived DAMPS engage with TLRs further amplifying production of NF-kappaB, cytokines and O and NS [4,50]. Hence, chronic inflammation and immune activation can be maintained and amplified by engagement of TLRs by DAMPS [4].

Chronically elevated levels of NF-kappaB, proinflammatory cytokines and O and NS, in turn, lead to a disruption of epithelial tight junctions in the intestine allowing translocation of gram-negative bacteria, containing lipopolysaccharides, into the circulation, which can further amplify the TLR-radical cycle by acting as a pathogen-associated molecular pattern (PAMP) [1]. Translocation of bacterial lipopolysaccharides (LPS) from the gut and engagement with TLRs, due to a state of increased intestinal permeability driven by the effector molecules of chronic inflammation is another cause of chronic immune activation that may play a role in major depression, CFS, neuro-inflammatory disorders and some systemic autoimmune disorders [6,7]. For example, further evidence of chronic immune activation in these neuroimmune and autoimmune illnesses is provided by data demonstrating TLR activation and upregulation in multiple sclerosis (MS) [51] and SLE [52].

Given the established association between chronic inflammation and the genesis of incapacitating fatigue [1], the TLR-radical cycle can potentially explain the development of incapacitating fatigue in patients suffering from these and other illnesses. This association may be explained by chronically increased levels of proinflammatory cytokines and reactive oxygen and nitrogen species (ROS/RNS) produced by the TLR-radical cycle upon stimulation by PAMPs and DAMPs [4]. We have reviewed previously that some proinflammatory cytokines, including IL-1β, TNF-α and IL-6, and increased O and NS processes may cause fatigue in some vulnerable individuals [1,4,6,7]. Mitochondrial dysfunction likely plays a major role in the progression of MS. Electron transport chain (ETC) complex I, complex III and complex IV activity is grossly reduced in normal appearing gray matter and in normal tissue within the motor cortex in patients suffering from this illness [53,54]. There is also direct evidence of globally impaired energy production and longitudinal depletion of ATP levels leads to increased levels of physical disability [55]. Multiple lines of evidence demonstrate the existence of mitochondrial dysfunction in many, but by no means all, patients afforded a diagnosis of CFS [56]. These abnormalities include loss of mitochondrial membrane integrity and oxidative corruption of translocatory proteins [57,58]. Other findings include abnormal muscle mitochondrial morphology and defective aerobic metabolism uncharacteristic of muscle disuse [59]. Several other teams have reported significant downregulation of oxidative phosphorylation in striated muscle [60,61]. Complex I deficiency is seen in the frontal cortex and substantia nigra of Parkinson’s disease patients [62], and this defect is also observed in peripheral tissues, such as skeletal muscle [63], strongly indicating a widespread reduction in complex I activity in Parkinson’s disease. Impaired complex III function has also been reported in the platelets and lymphocytes of patients with this illness [64]. There is also accumulating evidence that inflammation and subsequent mitochondrial dysfunction drive the symptoms of major depression [65,66]. Localized or global mitochondrial dysfunction is also an invariant feature of autoimmune diseases. Persistent mitochondrial membrane hyperpolarization and increased O and NS production combined with depleted levels of glutathione and ATP is an invariant characteristic of T cells in SLE [67,68]. The release of DAMPS into the systemic circulation, consequent to necrosis, acts as a mechanism by which localized mitochondrial pathology can lead to self-perpetuating systemic inflammation which, in turn, amplifies mitochondrial dysfunction in a vicious feed-forward loop [56,69]. The association between chronic oxidative stress, systemic inflammation and mitochondrial dysfunction and chronic oxidative stress is also firmly established in Sjogren's syndrome [70]. There is also evidence of widespread nitric oxide (NO)-induced inhibition of complex III and V of the ETC in patients with rheumatoid arthritis [71,72]. The causative role of chronic inflammation and oxidative stress and mitochondrial dysfunction is explained by the presence of elevated levels of ROS and RNS in such environments. These entities cause damage to proteins, DNA and lipid membranes [56]. NO and peroxynitrite have the capacity to inhibit crucial enzymes within the ETC and can inactivate crucial enzymes in the tricarboxylic acid cycle leading to, often critical, reductions in the generation of ATP [7]. Peroxynitrite, in particular, also has a destructive influence on the mitochondrial membrane leading to the loss of potential difference between the outer and inner membrane needed to manufacture ATP [7]. The products of lipid peroxidation driven by elevated levels of ROS are also toxic to mitochondrial membranes. It is noteworthy that inhibition of the ETC leads to the formation of even higher concentrations of oxygen radical species which, in turn, leads to further impairment of mitochondrial function [7]. Needless to say there are numerous studies demonstrating that the origin of severe intractable fatigue seen in people with syndromic mitochondrial diseases lies in mitochondrial pathology and depleted generation of ATP. The reader is referred to the work of [56] for further details.

**Figure 1:**
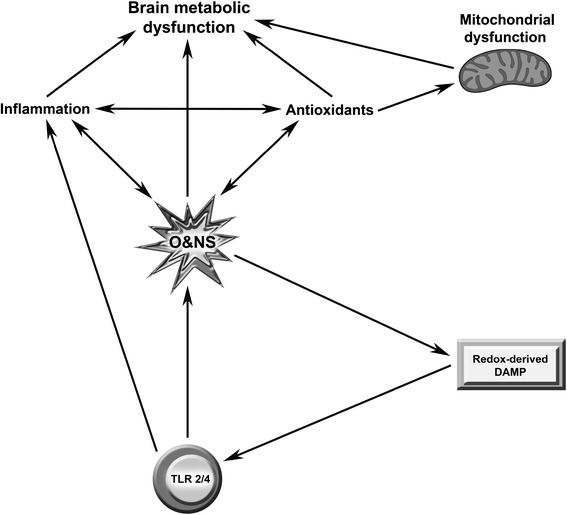
**Pathways associated with secondary fatigue.** Prolonged and or excessive stimulation of membrane bound Toll-like receptors (TLRs) results in the production of pro-inflammatory cytokines (PICs) and reactive oxygen and nitrogen species (ROS/RNS) at sufficiently high concentrations to cause macromolecule damage leading to the production of redox-derived damage-associated molecular patterns (DAMPs). The presence of such DAMPs leads to chronic engagement of TLRs and a spiraling, self-amplifying pattern of increasing ROS/RNS and PICs in a TLR radical cycle. Increasing levels of ROS/RNS damage mitochondrial lipids and proteins leading to dissipation of the mitochondrial membrane potential and inhibition of the electron transport chain. This leads to compromised oxidative phosphorylation and the production of ROS making another major contribution to the inflammatory milieu and another element in the development of a vicious spiral of bioenergetics decline. Elevated levels of PICs in the periphery activate microglia and astrocytes in the brain leading to the production of elevated PICs and ROS/RNS causing mitochondrial and metabolic dysfunction. This figure is original.

In this narrative review we will review the evidence pertaining to the genesis of intractable debilitating fatigue in multiple sclerosis, Parkinson’s disease, SLE, Sjogren’s disease, rheumatoid arthritis, major depression and CFS with a view of forming a conclusion as to whether such evidence justifies the viewpoint that the debilitating fatigue commonly suffered by those patients diagnosed with various illnesses is immune, inflammation or O and NS-mediated either directly or indirectly by causing abnormalities such as mitochondrial dysfunctions and central, neuropathological or functional processes [56,73-75]. These specific disorders were selected as examples along a spectrum of imbalance involving various degrees of activation of immune-inflammatory and O and NS pathways, and mitochondrial and brain metabolic dysfunctions in systemic auto-immune, immune-inflammatory and neurodegenerative disorders. Figure 1 shows the underlying processes and pathways associated with secondary fatigue, which we will discuss in the following sections.

### Multiple sclerosis

#### Fatigue in MS

Fatigue is recognized as one of the most disabling and common symptoms of MS affecting up to 80% of sufferers [17,76,77]. Numerous studies have demonstrated that the Expanded Disability Status Score (EDSS) correlates positively with patient self-reported fatigue scores using a variety of fatigue scales in patients with MS [78-81].

#### Immune activation, chronic inflammation and mitochondrial dysfunction

Chronic activation of the peripheral immune system is a characteristic observation in MS patients. Many studies report elevated levels of activated Th17 and Th1 T cells, and impaired function of regulatory T cells [17,82,83]. The evidence demonstrating an associative relationship between chronic activation of the immune system and the genesis of neuroinflammation is strong in MS due to the proven effectiveness of rituximab [84] and natalizumab [85], which are monoclonal antibodies which primarily target leucocytes but significantly reduce objective markers of disease activity in the central nervous system (CNS) [86]. It is also noteworthy that increased levels of TNF-α in the periphery are often predictive of the development of active disease. Peripheral TNF-α levels are also predictive of disability levels as estimated by the EDSS [87-89]. Peripheral levels of this and other cytokines correlate positively with fatigue severity which affects the vast majority of people with this illness [17,90-92]. TLR4 receptors are also upregulated in the brain and peripheral immune system in patients with MS [93-95]. There is also copious evidence indicating that chronic systemic inflammation and oxidative stress play a causative role in the etiopathogenesis of MS [96-98]. Elevated markers of chronic inflammation and oxidative stress are found in the brain, cerebrospinal fluid (CSF) and various blood compartments [82,99]. Oxidative stress levels increase quite dramatically during relapses but drop to barely detectable levels in patients during the remission phase [100]. It is also noteworthy that levels of chronic inflammation and oxidative stress in the CSF and blood correlate positively and significantly with disability levels as estimated by EDSS [101,102]. Finally, the extent of gadolinium-enhanced lesions appears to correlate significantly and positively with levels of oxidative stress [102].

It appears that although the genesis of pathology in early disease is mainly driven by inflammation [103], mitochondrial dysfunction likely plays a pivotal role in disease progression. Oxidative damage to mitochondrial DNA and impaired complex 1 activity is a characteristic finding in active MS lesions [104], but complex I, complex III and complex IV activity is also reduced in normal appearing gray matter and in normal tissue within the motor cortex [53,54,105].

The use of nuclear magnetic resonance (NMR) spectroscopy has found direct evidence of globally impaired energy production and increased lactate production in the CSF [106-108]. In a longitudinal study, progressive central depletion of ATP over a three year period correlated positively and significantly with increased indices of physical disability as measured by EDSS changes, which strongly suggests a global impairment of ATP synthesis in MS [108].

#### Neuroimaging and neuropathology

Until recently, all studies investigating the phenomena had failed to find any significant correlation between increasing self-reported fatigue during the performance of sustained cognitive tasks and changes in brain activity using any neuroimaging modality [109]. It has been argued that this situation has arisen because self-reported fatigue is not an objective or accurate indicator of cognitive performance in the first place [109]. However, the first evidence displaying a positive relationship between cognitive fatigue and changes in brain activity during a task was provided in a recent study [109]. While the relationship between self-reported fatigue and neuroimaging changes is still a matter of considerable debate, the positive association between changes in brain activity and objective measures of cognitive fatigue is generally accepted [110,111]. The bulk of evidence demonstrates that these changes in activity occur in several areas of the brain with most studies reporting this phenomenon in the basal ganglia and the prefrontal cortex [109]. Overall, the results of these studies have been interpreted as support for the hypothesis that the origin of fatigue seen in patients with MS and other neurological diseases arises as a result of failure of integrative processes within the basal ganglia which normally coordinate inputs from the limbic system and outputs to the motor cortex [109,112]. MS was once considered to be a disease of white matter but there is now overwhelming evidence that gray matter pathology occurs early in the disease often before the advent of white matter involvement [113,114]. Conventional magnetic resonance imaging (MRI) is of limited value in revealing gray matter pathology but newer MRI approaches based on FLAIR technology and NMR spectroscopy appear to display adequate sensitivity [114,115]. Gray matter atrophy occurs in very early stages of disease and is seen in people with clinically isolated syndrome (CIS) [115-117]. Indeed, this phenomenon is detected in people with first attack MS [118]. The extent of gray matter atrophy correlates significantly and positively with the degree of physical disability and cognitive impairment seen in many patients with this illness [119,120]. It is noteworthy that reduced gray matter perfusion is seen in very early disease without any loss of volume or other visible sign of gray matter (GM) pathology [121]. Cortical inflammation and metabolic abnormalities, such as reduced choline and N-acetyl aspartamine levels, are also evident in early MS without evidence of any kind of gray or white matter abnormalities [114,119,122]. Other studies, when viewed as a whole, have established a clear relationship between global or localized gray matter atrophy and hypoperfusion in the development of fatigue [123-126]. Other observations include an association between fatigue and glucose hypometabolism in the basal ganglia and frontal cortex [127-129] and a decreased N-acetyl aspartamine/creatine ratio in the basal ganglia, suggestive of gliosis [130].

Finally, Calabrese *et al*. reported a positive association between increased fatigue and widespread atrophy of the basal ganglia and prefrontal cortex [131]. It is tempting to speculate that these observations could arise from astrogliosis and underlying loss of astrocyte numbers and the normal regulatory functions of the surviving astrocyte population. Recent evidence indicates that reactive astrogliosis may play a major causative role in the development and progression of MS [132,133]. It is also worthy of note that astrocyte loss is a characteristic feature of this disease [134]. Protoplasmic astrocytes are primarily found in gray matter and form the vast bulk of cells located in this tissue [135]. These glial cells in particular have crucial roles in coordinating neurometabolic and neurovascular coupling and, hence, the delivery of oxygen and energy to neurons [136,137]. Given that astrocytes form the vast bulk of gray matter it seems likely that the loss of gray matter seen very early in the development of the disease is due to loss of astrocytes [138]. It is also interesting that the magnitude of gray matter loss correlates positively with severity of inflammation [138]. The presence of reactive astrogliosis would suggest that the regulatory performance of the remaining astrocytes could be compromised and, thus, would go some way to explaining the abnormalities in perfusion and glucose metabolism and the development of fatigue seen in these studies. This state of affairs could explain, in part, the regulatory dysfunction seen in the basal ganglia which seems to underpin the observations surrounding the changes in brain activity and the development of cognitive fatigue noted earlier.

### Chronic fatigue syndrome

#### Fatigue in chronic fatigue syndrome

Pathological levels of fatigue unrelated to activity and not relieved by rest is a mandatory requirement for a diagnosis of chronic fatigue syndrome under the current internationally accepted diagnostic guidelines [139]. The original diagnostic criteria contained another mandatory element, namely a clinical picture whereby the patient’s global symptoms represent a unitary illness with a single pathogenesis and pathophysiology. It is more likely that a diagnosis of CFS represents a spectrum of illnesses where different pathophysiological processes converge to produce a very similar phenotype [140]. Hence, any information regarding immune abnormalities, chronic inflammation, mitochondrial dysfunction and neuroimaging should be viewed with these issues in mind [141].

#### Immune activation, chronic inflammation and mitochondrial dysfunction

Numerous research teams have reported a wide range of peripheral immune abnormalities in people afforded a diagnosis of CFS [1,142,143]. The presence of circulating activated Th1, Th2 and Th17 T cells have all been detected. Recent evidence has challenged the view that people with CFS display immune abnormalities consistent with a Th2 pattern of T cell differentiation, and now data reveal that while some patients present with a Th2 profile and a preponderance of anti-inflammatory cytokine production, others present with a Th1 or possibly Th17 profile, with the synthesis of proinflammatory cytokines being dominant [144-146]. Elevated levels of TNF-α and IL-1B are, in fact, particularly commonplace observations in patients recruited into studies using the internationally agreed [139] diagnostic guidelines [144,147-151]. We have reviewed previously that patients with CFS and Myalgic Encephalomyelitis (ME) show different cytokine profiles, for example, a Th1-like pattern, with increased levels of IFN-γ, IL-2, IL-12 and IL-2 receptor, or a Th2-like pattern, with increased levels of IL-10, IL-4 and IL-5, or combinations thereof [1]. Two recent studies reported evidence of activated TLR4 receptors [152-154]. The causative relationship between chronic inflammation and the development of fatigue is perhaps strongest in patients afforded a diagnosis of CFS, with many studies demonstrating a significant positive correlation between surrogate markers of inflammation, oxidative stress and symptom severity [17,155-159]. Miwa and Fujita (2010) demonstrated that a rapid decline in inflammation and oxidative stress of patients corresponded with a decline in severity of fatigue and amelioration of their entire symptom profile [160]. Markers of chronic inflammation and oxidative imbalance have also been detected in skeletal muscle and levels of oxidative stress in this patient population correlated positively with objective measures of muscle fatigability [161]. Numerous authors have reported abnormalities consistent with mitochondrial dysfunction in patients afforded a diagnosis of CFS [56]. These abnormalities include loss of mitochondrial membrane integrity and oxidative corruption of translocatory proteins [57,58,162]. Other findings include abnormal muscle mitochondrial morphology and defective aerobic metabolism uncharacteristic of muscle disuse [59,163]. Several other teams utilizing 31-P NMR spectroscopy have reported significant down regulation of oxidative phosphorylation [60,61,164-167]. Other studies reported the presence of abnormal lactate responses to exercise indicative of a shift to glycolytic energy generation in at least some patients with a CFS diagnosis [168]. In a recent review, Filings and others [169] conclude that there was ample evidence of mitochondrial dysfunction and impaired bioenergetics performance in patients afforded a diagnosis of CFS, but once again it was confined to patients diagnosed according to internationally agreed criteria and not apparent in all patients [169]. Defects in oxidative phosphorylation and ATP generation have also been revealed in exercise testing with the pattern of physiological responses being characteristic of mitochondrial dysfunction [170]. Exercise performance was examined in a cohort of CFS patients and a loss in the linear relationship between heart rate and cardiac output and the dissipation of oxygen concentration gradient between venous and arterial blood characteristic of mitochondrial dysfunction was reported [171]. Finally, authors ultilizing NMR spectroscopy have reported that some patients with CFS display significantly elevated ventricular lactate levels, again suggestive of a shift towards aerobic glycolysis [159,172,173].

#### Neuroimaging and neuropathology

There is now considerable neuroimaging evidence demonstrating impaired blood flow in the cortex and cerebellum in many patients with a diagnosis of CFS [174-176]. Other studies report loss of gray matter volume [177-179]. Interestingly, this phenomenon has also been observed in patients given a primary diagnosis of fibromyalgia which is held by many to be an overlapping illness. Kuchina *et al*. reported that patients displayed levels of gray matter loss which were some three times greater than expected for their age [180]. Another study using 3-T voxel-based morphometry MRI reported reduced occipital lobe gray and white matter volume in the CFS group [181]. Cook and fellow workers, using functional MRI (fMRI) reported a significant positive association between perceived severity of fatigue and responsiveness in the cingulate frontal, temporal and cerebellar regions [182]. Another research team demonstrated impaired fMRI activation in the dorsolateral, dorsomedial and prefrontal cortices during a fatigue provocation task [183]. Glucose hypometabolism, especially in the prefrontal cortex, has also been demonstrated [184,185]. Finally Barden *et al.* [186] once again using 3 T MRI-based morphometric analysis reported evidence of astrocyte dysfunction and failure of autoregulatory mechanisms in patients in their trial cohort [186].

### Parkinson’s disease

#### Fatigue in Parkinson’s disease

Pathological fatigue, often described as a state of overwhelming exhaustion not necessarily related to physical effort, is recognized as a major, and possibly the most common, non-motor symptom of Parkinson’s disease [187,188] and often presents an insurmountable problem for patients and their caregivers [189,190]. Profound fatigue is experienced by some 82% of patients with advanced (HY stage 5) disease and the prevalence of fatigue increases with disease severity [191]. Although fatigue has been clearly established as an independent non-motor symptom of Parkinson’s disease, it is often confused with depression or excessive daytime sleepiness in clinical practice [189]. Some authors have actually adduced evidence indicating that fatigue could even be a pre-motor feature of Parkinson’s disease [192,193]. Schifitto *et al*. reported the presence of fatigue in just over a third of untreated non-depressed patients [194]. Furthermore, several other authors have reported that pathological levels of fatigue occur in non-depressed patients who are also untroubled by sleep problems [187,189].

#### Immune activation, inflammation and mitochondrial dysfunction

Numerous authors have reported that the serum and CSF of Parkinson’s disease patients contain elevated levels of activated CD4 and CD8 T cells and IL-1β, TNF-α, and IL-2 [195-199]. Increased frequencies of activated CD4^+^ T cells expressing the programmed death receptor Fas [198] and increased numbers of IFN-γ-producing Th1 cells, decreased numbers of IL-4-producing Th2 cells, and an overall decrease in CD4^+^CD25^+^ T cells have been found in the peripheral blood compartment of patients with this illness [200]. Studies have demonstrated that elevated peripheral cytokine production influences the progression of this illness. Parkinson patients display increased serum levels of TNF-α and TNF-α receptor 1 when compared to healthy control subjects, which makes an independent contribution to the pathogenesis of this illness [197,201,202]. It is also noteworthy that elevated plasma IL-6 concentrations significantly and positively correlate with increased risk of developing the illness [203].

#### Neuropathy and functional central processes

The increased frequencies of activated peripheral and memory T-cell subsets and activated T cells in the substantia nigra indicate the putative roles of T cells in the progression of Parkinson’s disease. There is also evidence that the balance of regulatory or effector T lymphocytes at inflammatory foci can either attenuate or exacerbate neuroinflammation and, hence, the subsequent development of neurodegeneration [13].

The intimate association between Parkinson’s disease and chronic inflammation has been revealed in different studies [204-208]. It is now recognized that chronic systemic inflammation plays a major role in the pathophysiology of Parkinson’s disease [209,210]. Nitrated proteins, DNA damage and lipid peroxidation bear testimony to the presence of elevated oxidative and nitrosative species [211,212]. The detection of extracellular HMGB1 and corrupted protein, DNA and lipid derived entities suggests substantial DAMP activity [213]. The weight of evidence indicates that the engagement of high-mobility group protein B1 (HMGB1) and alpha synuclein plays a major part in exacerbating the pathology of Parkinson’s disease [214,215]. Due to its modified conformation alpha synuclein behaves as a DAMP by activating TLR4 receptors on microglia resulting in the release of a plethora of neurotoxic entities, toxic molecules, including O and NS and proinflammatory cytokines and prostaglandin E2 (PGE2), thereby exacerbating neuro-inflammation [216,217].

Mitochondrial dysfunction in Parkinson’s disease in the shape of Complex I (CI) impairment has been suggested to be one of the fundamental causes of the illness [218,219]. This complex I deficiency is seen in the frontal cortex and substantia nigra in the patients [62], and in peripheral tissues, such skeletal muscle [220-222] and platelets [63,223,224], strongly indicating a widespread reduction in complex I activity in Parkinson’s disease. This defect is likely due to oxidative damage to complex 1 and possibly mis-assembly, as this latter phenomenon has been observed in isolated Parkinson’s disease brain mitochondria [225]. This complex I inhibition can induce the degeneration of neurons via a number of different mechanisms, such as excitotoxicity and increased oxidative stress [226]. A decrease in complex III function has also been reported in the platelets and lymphocytes of patients with this illness [64,223]. An association between the level of impairment of mitochondrial complex III assembly leading to a subsequent increase in ROS production and the development of Parkinson’s disease has also been reported [227]. This elevation in free radical production and release likely stems from the increased leakage of electrons from complex III. An alternative, but not mutually exclusive, explanation is that the inhibition of complex III assembly results in a severe reduction in the levels of functional complex I in mitochondria [228], again leading to an increase in ROS production via complex I deficiency. It is also noteworthy that the complex I and II electron acceptor ubiquinone is also reduced in the mitochondria of patients with Parkinson’s disease [229].

#### Neuroimaging and neuropathology

An almost bewildering array of neuroimaging abnormalities have been observed in patients with Parkinson’s disease and overall it is now clear that the various manifestations of the disease cannot be attributed to basal ganglia dysfunction alone [230,231]. Numerous studies employing voxel based morphometry have revealed a global pattern of gray matter loss and conformational abnormalities in Parkinson patients [232,233]. These gray matter changes are associated with cognitive and memory impairments which are seen in patients with very early disease [234,235]. Nagano-Saito and others reported that gray matter density displayed a positive and significant correlation in the dorsolateral prefrontal cortex and parahippocampal gyrus [236]. Loss of gray matter volume is apparent in treatment naive patients, once again bearing testimony to the existence of these abnormalities at the earliest stages of the disease [237]. The use of NMR spectroscopy has revealed neurometabolic abnormalities particularly a decrease in N-acetyl aspartate levels [238]. Finally, the use of the same technique has revealed the existence of widespread mitochondrial dysfunction in the brains of people with Parkinson’s disease even in the absence of any overt clinical manifestations [239]. Treatment naïve patients also display glucose hypometabolism in the dorsal pons, putamen and ventral thalamus [240-242]. Positron emission tomography (PET) imaging has revealed cortical hypometabolism in Parkinson’s disease. The severity and topography of glucose hypometabolism in the frontal and occipital cortex seen even in prodromal patients [243] intensifies and involves the lateral parietal and prefrontal cortices [242,244,245] and may also include the medial frontal and occipital regions [243,246] in patients with mild cognitive impairment (MCI). The severity and location of this hypometabolism may reflect the degree and extent of cognitive dysfunction [243,245,247,248]. The widespread cortical hypo-perfusion reported by many authors is also apparent at very early stages of disease and also appears to be related to the development of cognitive dysfunction [246,249,250].

### Major depressive disorder

#### Fatigue in depression

Fatigue of variable severity occurs in practically 100% of people with a diagnosis of depression [251,252]. It is worthy of note, however, that a systematic review reported that almost 80% of patients still experienced chronic debilitating levels of exhaustion following treatment of their depression [253]. This is perhaps to be expected given that several studies have now demonstrated that antidepressants have no positive modulatory effects on fatigue [254-257].

#### Immune activation, inflammation and mitochondrial dysfunction

The existence of increased levels of circulatory proinflammatory cytokines in these patients is now a textbook truism [20]. The picture regarding patterns of cytokine imbalance is complex with elevated levels of anti-inflammatory cytokines often reported [258]. There is copious evidence of chronically activated T cells with Th1, Th2 and Th17 patterns of differentiation [20,259,260]. It is worthy of note, however, that T cells appear to be dysfunctional, displaying an overall pattern of abnormalities consistent with a state of anergy [261]. Until recently, evidence of TLR activation in depression was limited to an animal model [262] but recently a study reported elevated levels of TLR4 in the brains of depressed patients displaying suicidal ideation [263]. Chronic systemic inflammation and oxidative stress play a major role in the etiology of depression [19,20]. Elevated levels of redox-damaged DAMPs, including oxidized low density lipoprotein, oxidized phospholipids, and malondialdehyde (MDA)-adducts are also consistently found in patients suffering from this illness [48]. Compromised epithelial barrier integrity is also a finding in depression and the resulting bacterial translocation into the systemic circulation is intimately involved in the pathogenesis of the disease [20,155]. Mitochondrial dysfunction affects neuronal function, synaptic plasticity, energy metabolism and neurotransmitter release and, hence, it is not surprising that there is increasing evidence that mitochondrial dysfunction and inflammation drive the symptoms of major depression [65,66]. Gardner and Boles highlighted the fact that research has failed to confirm a consistent relationship between serotonin levels and depression and that compromised bioenergetics should become a focus of research into the pathogenesis of the illness [264].

#### Neuroimaging and neuropathology

Hamilton and fellow workers reported the results of their meta-analysis of studies ultilizing various modalities of functional neuroimaging in patients with depression [265]. These authors concluded that a synthesis of the studies revealed a pattern of higher baseline neural activity in the pulvinar nucleus [265]. They further reported that studies ultilizing negative stimuli demonstrated a significantly greater neural response in certain areas of the brain, such as the amygdala, and lower responses in other regions, such as the prefrontal cortex, possibly indicating impaired contextual processing and reappraisal of visceral inputs [265]. In another meta-analysis, Kempton and others reported that patients with a diagnosis of depression and bipolar disorder displayed increased rates of hyperintensities in subcortical gray matter and increased volume of the lateral ventricles compared to healthy controls [266]. Interestingly, this meta-analysis also revealed distinct differences in neuroimaging abnormalities between depression and bipolar disorder, with the former having reduced rates of hyper-intensities in white matter and smaller basal ganglia and hippocampi compared to bipolar patients [266]. There is evidence that patients in a state of depression display reduced gray matter volume in the hippocampus compared to healthy controls or patients in remission [267]. Other investigators analyzing studies involving voxel based morphometric analysis have reported more widespread loss of gray matter in many different areas of the brain, especially in the prefrontal cortex [268-270]. It is noteworthy that gray matter reduction is evident in patients with first episode depression [271]. Impaired perfusion in frontotemporal regions has been reported [272] and a recent study has reported global cerebral hypoperfusion [273]. Interestingly, the degree of hypoperfusion in the prefrontal cortex correlates positively with the severity of depressive symptoms in patients with Alzheimers disease [274]. Another research group has recently reported that regional cerebral blood flow abnormalities in the prefrontal cortex and anterior cingulate cortices reverse during remission [275]. Glucose hypometabolism has been demonstrated in depressed patients both in the prefrontal cortex [276] and in several other regions [277]. An intriguing connection between glucose hypometabolism was proposed in a study by Hirono and others, who reported a positive significant association with the presence and severity of depressive symptoms in Alzheimer patients and decreased glucose metabolism in the frontal lobe [278]. Finally, the presence of activated microglia in patients suffering from depression has been established via the use of *in vivo* non-invasive neuroimaging [279].

### Systemic lupus erythematosus

#### Fatigue in SLE

Fatigue is an extremely common and disabling symptom affecting some 80% of patients with SLE [280]. Fatigue severity scores are significantly higher than population norms and similar to levels seen in patients with MS and Lyme disease [281,282]. Chronic debilitating fatigue is a major cause of morbidity in patients with SLE [283], that decreases quality of life [284-286] and increases work disability [287,288]. The aerobic capacity of patients with mild SLE is comparable to that observed in patients with severe cardiopulmonary disease [289-291]. Disease activity appears to be a major factor in the genesis of fatigue although this relationship is not evident in all studies [280,283,292,293].

#### Immune activation, inflammation and mitochondrial dysfunction

There is extensive evidence of activated T cells in the peripheral immune system of patients with SLE [294]. Elevated levels of proinflammatory cytokines play a key role in the pathophysiology of SLE [295]. Salbry *et al*. [296] reported a significant positive correlation between levels of TNF-α and IL-6 and objective markers of disease activity [296]. The weight of evidence indicates that significantly elevated levels of proinflammatory cytokines in the systemic circulation also plays a causative role in the development of systemic inflammation [297,298]. The presence of a chronic inflammatory state in people suffering from SLE has been reported by several research teams [28,299]. Wang and colleagues reported a significant positive correlation between elevated markers of O and NS with disease activity in this illness [300]. A range of TLRs are involved in initiating and maintaining the pathology of SLE, including TLR4, TLR3, TLR9 and TLR7 [301,302]. Impaired clearance of apoptopic cells is a pathological feature of SLE and, hence, the blebs and modified cellular contents act as autoantigens and are recognized by the immune system as DAMPS with the resultant activation of TLRs especially TLR4 [303,304]. The impaired clearance of these cells sets off a sequence of biochemical events allowing the escape of extramatrix debris once again acting as an autoantigen and recognized as a DAMP with the consequent activation of TLR4 and, indeed, a range of other TLRs as well [304]. Interestingly, polymorphisms in TLR4 (and CD14) genes are now thought to play a significant role in the etiopathogenesis of SLE. Persistent mitochondrial membrane hyperpolarization, increased O and NS production combined with depleted levels of glutathione and ATP is characteristic of T cells in SLE [67,68]. This environment sensitizes T cells towards necrotic cell death and the consequent release of DAMPS into the blood stream affords a mechanism by which localized mitochondrial pathology can lead to self-perpetuating systemic inflammation [69,305].

#### Neuroimaging and neurological abnormalities

Neurological symptoms in SLE are commonplace, affecting upwards of 80% of sufferers [32]. These neurological abnormalities occur even in the absence of the various systemic disease manifestations [306]. Voxel based morphometric analysis revealed widespread gray matter volume reduction in patients diagnosed with SLE [307-309]. Other studies have revealed the presence of white matter hyper-intensities, whose prevalence in an individual is predictive of disease progression [309-311]. The presence and severity of fatigue in patients with SLE is associated with white matter hyperintensities [312]. These authors reported that the White Matter Hyperintesity score correlated positively and significantly with fatigue severity [312]. The pathophysiology of ‘neuropsychiatric’ Lupus is mediated by cytokines, complement components and autoantibodies leading to the development of neuroinflammation and, ultimately, apoptosis of neurons and glial cells [313-316]. It is perhaps no surprise that the presence of activated microglia have been confirmed in patients with SLE [34].

### Sjogren's syndrome

#### Fatigue in Sjogren's syndrome

Fatigue and pain are, again, the most common extra-glandular symptoms of Sjogren's syndrome [317,318]. A total of 70% of patients with Sjogren’s syndrome suffer from fatigue and many patients state that fatigue is one of the most disabling symptoms of their disease [319]. There are a number of studies reporting a significant positive association between the severity of fatigue experienced by patients and various surrogate markers of disease activity [320-322]. The fatigue levels are associated with higher sicca symptoms, lower salivary volume, increased serum anti-Sjögren’s syndrome A antigen, immunoglobulin G (IgG) and proinflammatory cytokine levels [323]. Further evidence suggesting cytokine involvement in the genesis of fatigue was provided by Norheim and fellow workers who reported that patients’ fatigue levels were reduced by some 50% following blockade of IL-1β [324].

#### Immune activation, inflammation and mitochondrial dysfunction

Predictably there is copious evidence demonstrating the existence of a chronically activated innate immune system in patients diagnosed with this illness [325]. There is a wealth of data demonstrating disturbed cytokine networks [326], with cytokines secreted by activated Th1 and Th17 T cells being commonly detected in various blood compartments [327,328]. Epithelial cell activation leading to TLR upregulation is considered by many to be a pivotal early event in the pathogenesis of Sjogren's syndrome [329,330]. A range of TLRs, including TLR2, TLR3 and TLR4, are chronically up-regulated in sufferers of this illness [329,331]. Chronic systemic inflammation is an almost invariant finding in Sjogren's syndrome patients [332]. The existence of chronically elevated O and NS and subsequent oxidative stress has also been repeatedly demonstrated in patients with this disease [70,333]. The link between mitochondrial dysfunction and chronic oxidative stress is now firmly established in Sjogren's syndrome [70].

#### Neuroimaging and neurological abnormalities

A wide range of abnormalities in the central and peripheral nervous system occur in up to 70% of patients with Sjogren's syndrome, which may precede diagnosis in over 90% of cases [33,334,335]. Those interested in the details of these neurological abnormalities are invited to consult an excellent review by Tobon *et al*. [33]. There is some evidence that CNS pathology is immune mediated [336] and many patients display abnormalities on MRI with increased signaling intensity in T2 weighted images being the commonly noted finding [337,338]. These white matter hyperintensities (WMH) are indicative of widespread hypoperfusion [336,339-341]. Voxel based morphometry has once again revealed a global pattern of gray matter volume loss [340,342] and very recently loss of cerebral white matter was observed for the first time [343].

### Rheumatoid arthritis

#### Fatigue in rheumatoid arthritis

Patients with rheumatoid arthritis commonly complain of severe intractable fatigue with prevalence rates of up to 80% depending on definitions of fatigue used [344]. A study employing a fatigue measuring instrument reported that 40% of patients with rheumatoid arthritis experienced unremitting severe fatigue of the same level and pattern as fatigue experienced by patients with a diagnosis of chronic fatigue syndrome [345]. From a patient perspective fatigue is often described as extreme, unremitting and unrelated to activity and is associated with a failure to perform routine daily activities and non-refreshing sleep which, when considered together, are more debilitating than pain [346,347]. Reducing inflammation with disease modifiers significantly reduces fatigue [348]. Considerable evidence now exists demonstrating that the severity of fatigue experienced by patients suffering from this disease correlates significantly and positively with levels of disease activity [349,350].

#### Immune activation, inflammation and mitochondrial dysfunction

Numerous research teams have adduced evidence of a chronically activated immune system in rheumatoid arthritis patients as evidenced by significantly increased serum Th1, Th2 and Th17 cytokines [351-353]. Blockade of Th1 and Th17 cytokines can result in significant clinical benefit in patients with rheumatoid arthritis, strongly indicating their role as causative agents in the disease [354,355]. The frequency of Th17 T cells and associated cytokines strongly correlates with a poor prognosis which again suggests that these entities play a major causative role [356]. There is also good evidence that the use of biologic agents results in significant improvements in fatigue, strongly implicating elevated levels of these species in the genesis of intractable fatigue in patients with rheumatoid arthritis [357,358]. There is also considerable evidence demonstrating the activation and upregulation of TLRs in this disease with upregulated TLR2, TLR3 and TLR4 being commonplace findings [359-361]. Rheumatoid arthritis is recognized as being a systemic inflammatory condition [359] and chronic inflammation and accompanying oxidative stress play a causative role in the illness [362,363]. Perhaps unsurprisingly then, it has been demonstrated that levels of inflammation correlate positively with measures of disease activity [364]. The positive association between inflammation and fatigue genesis is evidenced by the fact that reducing inflammation with disease modifiers significantly reduces fatigue [348]. The effector molecules of chronic inflammation and oxidative stress can induce irreversible genetic changes and one such change, mutations in p53, has been suggested as a ‘turning point’ in converting a state of chronic inflammation into chronic disease [365]. There is evidence of somatic mutations in the mitochondrial DNA (mtDNA) within synoviocytes of rheumatoid arthritis patients which may confer immunogenicity on mtDNA derived proteins which consequently adopt the character of DAMPS and be one of such entities thought to play a major role in the etiopathogenesis of this disease [366]. A positive association has been reported in these cells between the extent of these mutations and the expression of cyclo-oxygenase 2 (COX-2), prostaglandin (PG)E2 and IL-8 [367]. The existence of these inflammatory markers is highly suggestive of NO-induced inhibition of complex III and V of the electron transport chain [72,368].

#### Neuroimaging and neuropathology

There is no direct evidence supporting the existence of chronically activated microglia and neuroinflammation in patients with rheumatoid arthritis, but neurological sequelae are commonplace and the role of chronic systemic inflammation in establishing such sequelae is accepted [35]. Wartoloska *et al*. reported widespread cortical atrophy in their patients with rheumatoid arthritis using unbiased voxel morphometric analysis and a pattern of increased gray matter density in subcortical areas notably the basal ganglia with the latter finding being suggestive of decreased dopamine levels [369]. An earlier MRI imaging study by Bekkelund and fellow workers also detected cortical atrophy in rheumatoid arthritis patients but only in those with longstanding disease [370].

### Cross-talk peripheral and CNS inflammation

**Figure 2:**
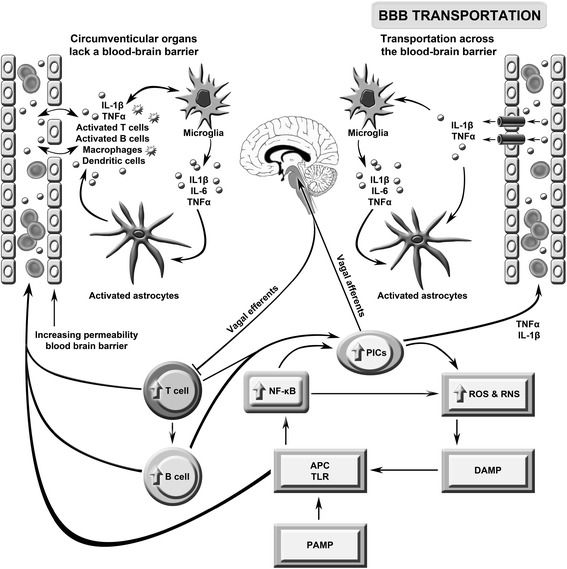
**This figure describes the putative role of immune brain communication in the pathogenesis of severe intractable fatigue.** Toll-like receptors (TLRs) on antigen presentation cells (APCs) may be activated by pathogen- or damage-associated molecular patterns (PAMPs/DAMPs) leading to the activation of nuclear factor-κB (NF-κB) and the subsequent upregulation of pro-inflammatory cytokines (PICs), including interleukin (IL)-1β, IL-6 and tumor necrosis factor (TNF)-α, and reactive oxygen and nitrogen species (ROS/RNS). These radical species may further damage macromolecules, increasing levels of redox-derived DAMPs which further engage TLRs in a self-sustaining cycle. PIC signals reach the brain via the afferent arm of the vagus nerve, engagement with transporters in the blood brain barrier (BBB) and passive diffusion. Inflammatory signaling from the periphery activates microglia which produce a range of neurotoxic molecules activating astrocytes causing a loss of brain homoeostasis and disruption of the BBB. The latter allows abnormally high numbers of activated T and B cells and macrophages to circulate between the periphery and the brain. This figure is original.

There is now copious evidence that chronic or intermittent inflammation, as observed in the abovementioned systemic disorders, can worsen or trigger neuroinflammatory or neurodegenerative processes via the induction of primed microglia [8,12]. Briefly, prolonged or intermittent peripheral inflammation and immune activation act to prime microglia which thereafter become exquisitely sensitive to future inflammatory stimuli [8]. Once microglia have achieved this sensitized status, subsequent peripheral inflammation and proinflammatory cytokine production mediated by a number of insults (for example, biotoxin exposure or pathogen invasion) provokes an exaggerated response from microglia and the production of excessive concentrations of neurotoxic molecules, such as nitric oxide, peroxinitrite, prostaglandins, cyclo-oxygenase 2 and cytokines [6,7]. The secretion of these neurotoxins and alarmins leads to the activation of astrocytes and the combined activation of these glial cells provokes dysregulation of brain homeostasis, development of chronic neuroinflammation and neurotoxicity. Both humoral and neuroendocrine routes mediate proinflammatory signaling to the brain. The neural route operates via the dorsal motor nucleus of the afferent vagus nerve [6]. The humoral route is facilitated by circulating proinflammatory cytokines that communicate their presence to the brain via direct and indirect routes. Such pathways involve engagement with specific transporters in the blood brain barrier (BBB), the activation of endothelial cells and macrophages, creating a mirror pattern of production on the adluminal side of the BBB, and passive diffusion into areas of the brain lacking a functional BBB (for example, circumventricular organs) and thereafter into the glial limitans [1]. The cumulative effects of proinflammatory cytokines and activated astrocytes cause disruption of the BBB allowing abnormally high numbers of activated T cells and B-cells to circulate between the peripheral immune system and the brain, acting as more channels of communication between the peripheral and central immune system [13]. It should be noted that cytokines are able to diffuse from the CNS into the bloodstream as well [13]. Finally, the presence of proinflammatory cytokines in the brain activates the hypothalamus instigating the cholinergic anti-inflammatory pathway designed to terminate the immune response [1,6]. These processes are depicted in Figure 2.

### ASIA syndrome and sex effects

All disorders reviewed here, except Parkinson’s disorder, are more frequent in women than in men. For example, in patients with rheumatoid arthritis a four to five greater incidence is found in women than in men when less than 50 years old, whereas these differences are less pronounced in 60- to 70-year old individuals. The female predilection is also observed in depression, CFS, MS, Sjogren’s syndrome and systemic lupus erythematosus [371-375]. In Parkinson’s disorder the male/female incidence rate ratio is 1.6 to 1 [376]. One main difference between Parkinson’s disease and the other disorders discussed here is that the autoimmune component is less pronounced in Parkinson’s disease. An increased incidence rate in women is observed in most autoimmune disorders [371]. Nevertheless, also in Parkinson’s disease autoantibodies are observed and they are associated with specific symptom profiles, including depression [377]. It is argued that these sex-related differences in incidence may be explained by endogenous sex-hormones.

Estrogen, progesterone and testosterone play important immunomodulatory roles and influence the quantity and pattern of cytokine secretion by antigen presentation cells and T lymphocytes and immunoglobulin production by B cells. Sex hormones also regulate the Th1/Th2 balance of the immune system, the production of regulatory T cells and the functionality of granulocytes and natural killer cells [378,379]. An interested reader is referred to an excellent review by [380] for a detailed consideration of the mechanistic effects of sex hormones on individual classes of immune cells. In the light of the discussion above, it also seems noteworthy that estrogen is neuroprotective in many animal models of neuroimmune and neurodegenerative disorders essentially by down regulating the expression of neuroinflammatory genes in glial cells, such as those coding for elements of the complement system, proinflammatory cytokines and TLRs [381]. Thus, excessive estrogens but less androgens may favor activation of B cells, a Th2-like response and increased numbers of autoimmune cells and, thus, autoimmune responses [371]. Nevertheless, the precise effects of sex- or gender-related factors on the increased incidence of autoimmune-related disorders has remained elusive. Future research should delineate not only sex but also gender-related effects according to the gendered innovations approach [382].

These parameters and elevated number of circulating T cells seen in premenopausal women may be one reason for the powerful prolonged activation of inflammatory pathways and adverse reactions to aluminum adjuvants seen in women following administration of a range of vaccines [383,384]. The engagement of TLR receptors by aluminum, as well as the activation of the NLP3 inflammasome, could create a state of chronic inflammation and oxidative stress in a person with functional polymorphisms in immune genes as discussed above and, hence, could be a cause of Autoimmune Inflammatory Syndrome Induced by Adjuvants (ASIA), alternatively known as Schoenfield’s Syndrome [385-387]. The activation of TLR4 by silicon [388] could also explain the connection of this element with the development of ASIA and the chronic activation of TLRs can potentially explain many environmental contributions to the ‘mosaic of autoimmunity’ [389].

Sex effects may also determine responsivity to drug therapy as, for example, in MS. Thus, postmenopausal women are poorer responders to rituximab than men of the same age [390,391]. This might seem a little counter intuitive from the frame of reference that rituximab exerts its effects mainly on the B cell population and that B cell levels do not appear to differ in postmenopausal women and age equivalent men to any significant extent [392]. However rituximab also exerts modulatory effects on the T cell compartment [393]. Numerous researchers have reported that the clinical benefits seen following the use of rituximab in rheumatoid arthritis and other autoimmune conditions are associated with the antibody’s capacity to increase the expression of FOXP3 [394], suppress the expression of retanoic acid-like orphan receptors ultimately suppressing the production of Th17 T cells and IL-17 [395] and reducing the expression of cytokines by Th1, Th2 and Th17 T cells [396]. It is possible that the Th2 shift in the immune system seen in postmenopausal women negates the benefits of rituximab on a Th1/Th17 biased immune system [392]. The positive benefits of rituximab and natalizumab on MS [84,85] is probably most easily explained by the modulatory effects of rituximab and, likely, natalizumab on the T cell compartment as well as their well-documented effects on B cell depletion.

## Summary and conclusion

**Figure 3:**
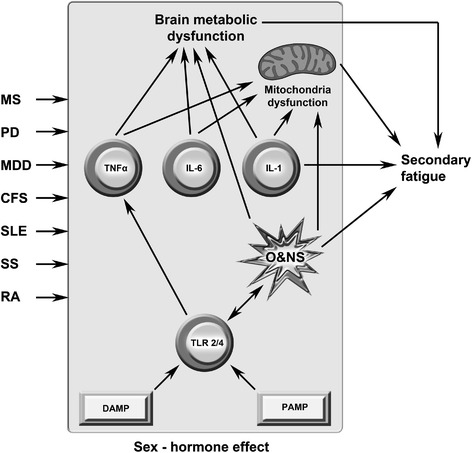
**This figure depicts shared pathways in the illnesses described in this paper that may cause secondary fatigue.** Activation of Toll-like receptors 2/4 (TLR2/4) by pathogen- and damage-associated molecular patterns (PAMPs/DAMPs) leads to the production of interleukin (IL)-1β, tumor necrosis factor (TNF)-α and IL-6 together with elevated levels of reactive oxygen and nitrogen species (ROS/RNS). IL-1β is a primary source of secondary fatigue and together with the other cytokines acts as a secondary source of fatigue via the inhibition of mitochondrial performance and the provocation of metabolic dysfunction in the brain via the activation of astrocytes and microglia. ROS/RNS can also be a primary cause of secondary fatigue by damaging lipids and proteins which are essential for the performance of mitochondria and inhibiting the electron transport chain. These actions lead to impaired mitochondrial performance which is also a source of fatigue in a similar manner as found in syndromic mitochondrial diseases. This figure is original.

Figure 3 shows a diagram illustrating the causal links being described in the above sections synthesizing the significant pathways that lead to secondary fatigue in these different neurodegenerative and systemic (auto)immune disorders. There is clear evidence of a positive relationship between fatigue severity and levels of disability in MS. It is of interest that levels of peripheral inflammation, oxidative stress and TNF-α also display a positive correlation with objective markers of disease activity and disability levels and that levels of proinflammatory cytokines correlate positively with levels of fatigue. The existence of gray matter atrophy before the advent of white matter abnormalities, and the existence of metabolic abnormalities before the advent of gray matter pathology, rather argues against the proposition that the chronic peripheral immune activation and oxidative stress seen in early disease is secondary to the release of inflammatory mediators from the CNS. These observations, coupled with data demonstrating that the severity of neuro-inflammation depends on the level of peripheral immune activation and that inflammation drives the development of disease, emphasizes the likely causative role of peripheral pathology. The strong association between the severity of fatigue and disability and the level and geographical distribution of glucose hypometabolism and gray matter hypoperfusion strongly indicates that these elements are driven by generic rather than disease specific pathology. These kinds of generic abnormalities are also evident in Parkinson’s Disease where peripheral immune activation, oxidative stress, GM atrophy and widespread glucose hypometabolism are all evidenced in the very earliest stages of disease development. It is also noteworthy that the prevalence of severe intractable fatigue increases with the degree of disease progression and that the degree of peripheral inflammation and levels of proinflammatory cytokines are predictive of disease development and severity. When viewed as a whole these observations also support the view that severe intractable fatigue results from processes which are not disease specific but involved in disease pathogenesis. The existence of chronic peripheral inflammation and immune activation together with GM atrophy and glucose hypometabolism in patients with first episode depression is now a textbook truism. Interestingly, the pattern of neuroimaging abnormalities and GM pathology appears to be quite distinct from that seen in patients with neuroimmune and autoimmune diseases for reasons which are not yet clear. This pattern of peripheral inflammation and immune activation is also found in autoimmune diseases with levels of oxidative stress and proinflammatory cytokines having a causative role in the pathophysiology of SLE and displaying positive correlations with objective markers of disease severity. This is also true of patients with Sjogren's syndrome where objective markers of disease activity are reduced by cytokine blockade. There is also evidence demonstrating that the severity of fatigue is associated with the degree of white matter hyperintensities in people with SLE and evidence that the neuropathology in Sjogren's syndrome is immune mediated. The widespread mitochondrial dysfunction seen in people with autoimmune diseases could also make a significant contribution to the development of fatigue. Widespread mitochondrial dysfunction, in otherwise normal tissue, is also seen in patients with MS, Parkinson’s disease and in many patients with apparently idiopathic fatigue. Given that many such patients also display evidence of peripheral immune activation, oxidative stress, gray matter pathology, glucose hypometabolism, hypoperfusion and metabolic abnormalities in the prefrontal cortex, basal ganglia and elsewhere, it would seem reasonable to investigate all such patients for the presence of these abnormalities. Standard MRI is unlikely to be helpful but other approaches discussed in the main body combined with serum measures of immune activation and oxidative stress may well bear fruit.

As these mechanisms are extensively inter-related, it should be underscored that without a solid prospective timeline and known systems biomedicine, it has remained difficult to distinguish causation from association. Therefore, future research should delineate: 1) the overwhelmingly complex and dynamic interactions between these different pathways and the intracellular networks that modulate them; and 2) the multifactorial triggers that cause secondary fatigue by activating the networks/pathways in those disorders, including viral and bacterial infections, bacterial translocation, psychosocial stressors, exposure to adjuvants, nicotine dependence, sex- and gender-related factors, and so on. Towards this end, a systems biomedicine approach is essential to delineate the genetic and molecular signature of fatigue in these disorders and the non-linear interactions between the many pathways, networks, and trigger and genetic factors that underpin secondary fatigue.

Multi-targeting these interlinked dysfunctions may show benefit in these diseases. For example, a number of antioxidant compounds have demonstrated efficacy in modifying pathways leading to chronic inflammation, oxidative stress and immune dysregulation at relatively high doses for a long duration [7]. N-acetyl-cysteine is an example of a multi-target therapeutic approach having the capacity to decrease the levels of ROS/RNS, increase the levels of cellular antioxidants, such as reduced glutathione, and normalize the production of proinflammatory cytokines and immune cell functions [397]. This supplement has demonstrated the capacity to improve fatigue and disease activity in SLE, CFS and major and bipolar depression [7,398]. Omega-3 polyunsaturated fatty acids (PUFAs) and zinc are also very effective antioxidants and anti-inflammatory compounds and supplementation has produced clinical benefit in patients diagnosed with depression and chronic fatigue syndrome [7,399,400]. Omega-3 PUFAs also show a clinical efficacy in SLE and rheumatoid arthritis [398,401,402]. Curcumin, another nutraceutical with anti-inflammatory and antioxidative effects, is useful in the treatment of depression and rheumatoid arthritis [403,404]. Coenzyme Q10 is another powerful antioxidant and anti-inflammatory compound which also has positive effects on mitochondrial function and which displays disease modifying effects in Parkinson’s disease and produced clinical benefit in patients with a diagnosis of CFS [56]. Other approaches aimed at upregulating antioxidant defenses include N acetylcysteine, methylfolate and dimethyl fumarate, with the latter displaying disease modifying properties in MS [140]. Methylfolate produces a similar quantum of benefit in MDD as antidepressants and can often be effective in treatment-resistant depression [140].

It is concluded that there are sufficient robust multiple lines of evidence to support the proposition that the severe fatigue and profound disability experienced by people with the neurodegenerative, neuro-immune and autoimmune diseases discussed here is largely driven by peripheral immune activation and systemic inflammation either directly or indirectly by inducing mitochondrial damage.

## References

[1] Morris G, Anderson G, Galecki P, Berk M, Maes M (2013). A narrative review on the similarities and dissimilarities between myalgic encephalomyelitis/chronic fatigue syndrome (ME/CFS) and sickness behavior. BMC Med.

[2] Norheim K, Jonsson G, Omdal R (2011). Biological mechanisms of chronic fatigue. Rheumatology.

[3] Jialal I, Kaur H, Devaraj S (2014). Toll-like receptor status in obesity and metabolic syndrome: a translational perspective. J Clin Endocrinol Metab.

[4] Lucas K, Maes M (2013). Role of the Toll Like receptor (TLR) radical cycle in chronic inflammation: possible treatments targeting the TLR4 pathway. Mol Neurobiol.

[5] Fernandez-Gonzalo R, De Paz JA, Rodriguez-Miguelez P, Cuevas MJ, González-Gallego J (2012). Effects of eccentric exercise on toll-like receptor 4 signaling pathway in peripheral blood mononuclear cells. J Appl Physiol.

[6] Morris G, Maes M (2013). A neuro-immune model of Myalgic Encephalomyelitis/Chronic fatigue syndrome. Metab Brain Dis.

[7] Morris G, Maes M (2014). Oxidative and nitrosative stress and immune-inflammatory pathways in patients with Myalgic Encephalomyelitis (ME)/Chronic Fatigue Syndrome (CFS). Curr Neuropharmacol.

[8] Perry VH, Cunningham C, Boche D (2002). Atypical inflammation in the central nervous system in prion disease. Curr Opin Neurol.

[9] Perry VH (2004). The influence of systemic inflammation on inflammation in the brain: implications for chronic neurodegenerative disease. Brain Behav Immun.

[10] Londoño D, Cadavid D (2010). Bacterial lipoproteins can disseminate from the periphery to inflame the brain. Am J Pathol.

[11] Perry VH (2010). Contribution of systemic inflammation to chronic neurodegeneration. Acta Neuropathol.

[12] Perry VH, Nicoll JA, Holmes C (2010). Microglia in neurodegenerative disease. Nat Rev Neurol.

[13] Mosley RL, Hutter-Saunders JA, Stone DK, Gendelman HE (2012). Inflammation and adaptive immunity in Parkinson’s disease. Cold Spring Harb Perspect Med.

[14] Holmes C, Cunningham C, Zotova E, Woolford J, Dean C, Kerr S (2009). Systemic inflammation and disease progression in Alzheimer disease. Neurology.

[15] Heesen C, Schulz KH, Fiehler J, Von der Mark U, Otte C, Jung R (2010). Correlates of cognitive dysfunction in multiple sclerosis. Brain Behav Immun.

[16] Arai H, Furuya T, Mizuno Y, Mochizuki H (2006). Inflammation and infection in Parkinson’s disease. Histol Histopathol.

[17] Morris G, Maes M (2013). Myalgic encephalomyelitis/chronic fatigue syndrome and encephalomyelitis disseminata/multiple sclerosis show remarkable levels of similarity in phenomenology and neuroimmune characteristics. BMC Med.

[18] Beiske AG, Svensson E (2010). Fatigue in Parkinson’s disease: a short update. Acta Neurol Scand Suppl.

[19] Berk M, Williams L, Jacka F, O’Neil A, Pasco J, Moylan S (2013). So depression is an inflammatory disease, but where does the inflammation come from?. BMC Med.

[20] Maes M, Berk M, Goehler L, Song C, Anderson G, Galecki P (2012). Depression and sickness behavior are Janus-faced responses to shared inflammatory pathways. BMC Med.

[21] Kreisel T, Frank M, Licht T, Reshef R, Ben-Menachem-Zidon O, Baratta MV (2014). Dynamic microglial alterations underlie stress-induced depressive-like behavior and suppressed neurogenesis. Mol Psychiatry.

[22] Steiner J, Walter M, Gos T, Guillemin G, Bernstein H, Sarnyai Z (2011). Severe depression is associated with increased microglial quinolinic acid in subregions of the anterior cingulate gyrus: evidence for an immune-modulated glutamatergic neurotransmission. J Neuroinflammation.

[23] Segal B, Thomas W, Rogers T, Leon JM, Hughes P, Patel D (2008). Prevalence, severity, and predictors of fatigue in subjects with primary Sjögren’s syndrome. Arthritis Rheum.

[24] Ahn GE, Ramsey-Goldman R (2012). Fatigue in systemic lupus erythematosus. Int J Clin Rheumtol.

[25] Hewlett S, Ambler N, Almeida C, Cliss A, Hammond A, Kitchen K (2011). Self-management of fatigue in rheumatoid arthritis: a randomised controlled trial of group cognitive-behavioural therapy. Ann Rheum Dis.

[26] Sattar N, McCarey D, Capell H, McInnes I (2003). Explaining how a “high-grade” systemic inflammation accelerates vascular risk in rheumatoid arthritis. Circulation.

[27] Ku I, Imboden J, Hsue P, Ganz P (2009). Rheumatoid arthritis: model of systemic inflammation driving atherosclerosis. Circ J.

[28] Munoz L, Janko C, Grossmayer G, Frey B, Voll R, Kern P (2009). Remnants of secondarily necrotic cells fuel inflammation in systemic lupus erythematosus. Arthritis Rheum.

[29] Lee HM, Sugino H, Nishimoto N (2010). Cytokine networks in systemic lupus erythematosus. J Biomed Biotechnol.

[30] Sisto M, Lisi S, Ingravallo G, Lofrumento D, D’Amore M, Ribatti D (2014). Neovascularization is prominent in the chronic inflammatory lesions of Sjögren’s syndrome. Int J Exp Pathol.

[31] Lisi S, Sisto M, D’Amore M, Lofrumento D, Ribatti D (2013). Emerging avenues linking inflammation, angiogenesis and Sjögren’s syndrome. Cytokine.

[32] Muscal E, Brey R (2010). Neurological manifestations of systemic lupus erythematosus in children and adults. Neurol Clin.

[33] Tobón G, Pers J, Devauchelle-Pensec V, Youinou P (2012). Neurological disorders in primary Sjögren’s syndrome. Autoimmune Dis.

[34] Meszaros Z, Perl A, Faraone S (2012). Psychiatric symptoms in systemic lupus erythematosus: a systematic review. J Clin Psychiatry.

[35] Ramos-Remus C, Duran-Barragan S, Castillo-Ortiz J (2012). Beyond the joints: neurological involvement in rheumatoid arthritis. Clin Rheumatol.

[36] Alvarez-Lafuente R, De las Heras V, Bartolomé M, Picazo JJ, Arroyo R (2004). Relapsing-remitting multiple sclerosis and human herpesvirus 6 active infection. Arch Neurol.

[37] Akhyani N, Berti R, Brennan MB, Soldan SS, Eaton JM, McFarland HF (2000). Tissue distribution and variant characterization of human herpesvirus (HHV)-6: increased prevalence of HHV-6A in patients with multiple sclerosis. J Infect Dis.

[38] Goldman S (2014). Environmental toxins and Parkinson’s disease. Annu Rev Pharmacol Toxicol.

[39] Khansari N, Shakiba Y, Mahmoudi M (2009). Chronic inflammation and oxidative stress as a major cause of age-related diseases and cancer. Recent Pat Inflamm Allergy Drug Discov.

[40] Tabruyn SP, Mémet S, Avé P, Verhaeghe C, Mayo KH, Struman I (2009). NF-kappaB activation in endothelial cells is critical for the activity of angiostatic agents. Mol Cancer Ther.

[41] Schmidt C, Peng B, Li Z, Sclabas GM, Fujioka S, Niu J (2003). Mechanisms of proinflammatory cytokine-induced biphasic NF-kappaB activation. Mol Cell.

[42] Sultani M, Stringer AM, Bowen JM, Gibson RJ (2012). Anti-inflammatory cytokines: important immunoregulatory factors contributing to chemotherapy-induced gastrointestinal mucositis. Chemother Res Pract.

[43] Nakata S, Tsutsui M, Shimokawa H, Yamashita T, Tanimoto A, Tasaki H (2007). Statin treatment upregulates vascular neuronal nitric oxide synthase through Akt/NF-kappaB pathway. Arterioscler Thromb Vasc Biol.

[44] Anrather J, Racchumi G, Iadecola C (2006). NF-kappaB regulates phagocytic NADPH oxidase by inducing the expression of gp91phox. J Biol Chem.

[45] Sonis ST (2007). Pathobiology of oral mucositis: novel insights and opportunities. J Support Oncol.

[46] Sonis ST (2004). A biological approach to mucositis. J Support Oncol.

[47] Morgan MJ, Liu ZG (2011). Crosstalk of reactive oxygen species and NF-κB signaling. Cell Res.

[48] Maes M, Kubera M, Obuchowiczwa E, Goehler L, Brzeszcz J (2011). Depression’s multiple comorbidities explained by (neuro)inflammatory and oxidative & nitrosative stress pathways. Neuro Endocrinol Lett.

[49] Maes M, Mihaylova I, Leunis JC (2006). Chronic fatigue syndrome is accompanied by an IgM-related immune response directed against neopitopes formed by oxidative or nitrosative damage to lipids and proteins. Neuro Endocrinol Lett.

[50] Kuper H, Adami HO, Trichopoulos D (2000). Infections as a major preventable cause of human cancer. J Intern Med.

[51] Miranda-Hernandez S, Baxter AG (2013). Role of toll-like receptors in multiple sclerosis. Am J Clin Exp Immunol.

[52] Horton C, Pan Z, Farris A. Targeting Toll-like receptors for treatment of SLE. Mediators Inflamm. 2010; 2010. doi:10.1155/2010/498980. 10.1155/2010/498980 PMC2945668 20886024

[53] Mahad DJ, Ziabreva I, Campbell G, Lax N, White K, Hanson PS (2009). Mitochondrial changes within axons in multiple sclerosis. Brain.

[54] Dutta R, McDonough J, Yin X, Peterson J, Chang A, Torres T (2006). Mitochondrial dysfunction as a cause of axonal degeneration in multiple sclerosis patients. Ann Neurol.

[55] Lazzarino G, Amorini AM, Eikelenboom MJ, Killestein J, Belli A, Di Pietro V (2010). Cerebrospinal fluid ATP metabolites in multiple sclerosis. Mult Scler.

[56] Morris G, Maes M (2014). Mitochondrial dysfunctions in myalgic encephalomyelitis/chronic fatigue syndrome explained by activated immuno-inflammatory, oxidative and nitrosative stress pathways. Metab Brain Dis.

[57] Booth NE, Myhill S, McLaren-Howard J (2012). Mitochondrial dysfunction and the pathophysiology of Myalgic Encephalomyelitis/Chronic Fatigue Syndrome (ME/CFS). Int J Clin Exp Med.

[58] Myhill S, Booth NE, McLaren-Howard J (2013). Targeting mitochondrial dysfunction in the treatment of Myalgic Encephalomyelitis/Chronic Fatigue Syndrome (ME/CFS) - a clinical audit. Int J Clin Exp Med.

[59] Behan WM, McDonald M, Darlington LG, Stone TW (1999). Oxidative stress as a mechanism for quinolinic acid-induced hippocampal damage: protection by melatonin and deprenyl. Br J Pharmacol.

[60] Jones DE, Hollingsworth KG, Taylor R, Blamire AM, Newton JL (2010). Abnormalities in pH handling by peripheral muscle and potential regulation by the autonomic nervous system in chronic fatigue syndrome. J Intern Med.

[61] Hollingsworth KG, Jones DE, Taylor R, Blamire AM, Newton JL (2010). Impaired cardiovascular response to standing in chronic fatigue syndrome. Eur J Clin Invest.

[62] Parker WD, Parks JK, Swerdlow RH (2008). Complex I deficiency in Parkinson’s disease frontal cortex. Brain Res.

[63] Blake C, Spitz E, Leehey M, Hoffer B, Boyson S (1997). Platelet mitochondrial respiratory chain function in Parkinson’s disease. Mov Disord.

[64] Shinde S, Pasupathy K (2006). Respiratory-chain enzyme activities in isolated mitochondria of lymphocytes from patients with Parkinson’s disease: preliminary study. Neurol India.

[65] Tobe EH (2013). Mitochondrial dysfunction, oxidative stress, and major depressive disorder. Neuropsychiatr Dis Treat.

[66] Manji H, Kato T, Di Prospero NA, Ness S, Beal MF, Krams M (2012). Impaired mitochondrial function in psychiatric disorders. Nat Rev Neurosci.

[67] Perl A, Hanczko R, Doherty E (2012). Assessment of mitochondrial dysfunction in lymphocytes of patients with systemic lupus erythematosus. Methods Mol Biol.

[68] Perl A, Nagy G, Gergely P, Puskas F, Qian Y, Banki K (2004). Apoptosis and mitochondrial dysfunction in lymphocytes of patients with systemic lupus erythematosus. Methods Mol Med.

[69] Nagy G, Koncz A, Fernandez D, Perl A (2007). Nitric oxide, mitochondrial hyperpolarization, and T cell activation. Free Radic Biol Med.

[70] Pagano G, Castello G, Pallardó FV (2013). Sjøgren’s syndrome-associated oxidative stress and mitochondrial dysfunction: prospects for chemoprevention trials. Free Radic Res.

[71] Cillero-Pastor B, Eijkel GB, Kiss A, Blanco FJ, Heeren RM (2013). Matrix-assisted laser desorption ionization-imaging mass spectrometry: a new methodology to study human osteoarthritic cartilage. Arthritis Rheum.

[72] Abramson SB (2008). Nitric oxide in inflammation and pain associated with osteoarthritis. Arthritis Res Ther.

[73] Rose S, Frye RE, Slattery J, Wynne R, Tippett M, Melnyk S (2014). Oxidative stress induces mitochondrial dysfunction in a subset of autistic lymphoblastoid cell lines. Transl Psychiatry.

[74] Imaizumi Y, Okada Y, Akamatsu W, Koike M, Kuzumaki N, Hayakawa H (2012). Mitochondrial dysfunction associated with increased oxidative stress and α-synuclein accumulation in PARK2 iPSC-derived neurons and postmortem brain tissue. Mol Brain.

[75] Cui H, Kong Y, Zhang H (2012). Oxidative stress, mitochondrial dysfunction, and aging. J Signal Transduct.

[76] Lapierre Y, Hum S (2007). Treating fatigue. Int MS J.

[77] Bakshi R (2003). Fatigue associated with multiple sclerosis: diagnosis, impact and management. Mult Scler.

[78] Patrick E, Christodoulou C, Krupp LB, New York State MS Consortium (2009). Longitudinal correlates of fatigue in multiple sclerosis. Mult Scler.

[79] Flachenecker P, Kümpfel T, Kallmann B, Gottschalk M, Grauer O, Rieckmann P (2002). Fatigue in multiple sclerosis: a comparison of different rating scales and correlation to clinical parameters. Mult Scler.

[80] Iriarte J, Subira ML, Castro P (2000). Modalities of fatigue in multiple sclerosis: correlation with clinical and biological factors. Mult Scler.

[81] Tellez N, Rio J, Tintoré M, Nos C, Galán I, Montalban X (2005). Does the Modified Fatigue Impact Scale offer a more comprehensive assessment of fatigue in MS?. Mult Scler.

[82] Ortiz GG, Pacheco-Moisés FP, Bitzer-Quintero OK, Ramírez-Anguiano AC, Flores-Alvarado LJ, Ramírez-Ramírez V (2013). Immunology and oxidative stress in multiple sclerosis: clinical and basic approach. Clin Dev Immunol.

[83] Nakamura M, Matsuoka T, Chihara N, Miyake S, Sato W, Araki M (2014). Differential effects of fingolimod on B-cell populations in multiple sclerosis. Mult Scler.

[84] Hauser SL, Waubant E, Arnold DL, Vollmer T, Antel J, Fox RJ (2008). B-cell depletion with rituximab in relapsing-remitting multiple sclerosis. N Engl J Med.

[85] Polman CH, O’Connor PW, Havrdova E, Hutchinson M, Kappos L, Miller DH (2006). A randomized, placebo-controlled trial of natalizumab for relapsing multiple sclerosis. N Engl J Med.

[86] Romme Christensen J, Börnsen L, Hesse D, Krakauer M, Sørensen PS, Søndergaard HB (2012). Cellular sources of dysregulated cytokines in relapsing-remitting multiple sclerosis. J Neuroinflammation.

[87] Beck J, Rondot P, Catinot L, Falcoff E, Kirchner H, Wietzerbin J (1988). Increased production of interferon gamma and tumor necrosis factor precedes clinical manifestation in multiple sclerosis: do cytokines trigger off exacerbations. Acta Neurol Scand.

[88] Maimone D, Gregory S, Arnason BG, Reder AT (1991). Cytokine levels in the cerebrospinal fluid and serum of patients with multiple sclerosis. J Neuroimmunol.

[89] Navikas V, Link H (1996). Review: cytokines and the pathogenesis of multiple sclerosis. J Neurosci Res.

[90] Gold SM, Krüger S, Ziegler KJ, Krieger T, Schulz KH, Otte C (2011). Endocrine and immune substrates of depressive symptoms and fatigue in multiple sclerosis patients with comorbid major depression. J Neurol Neurosurg Psychiatry.

[91] Heesen C, Nawrath L, Reich C, Bauer N, Schulz KH, Gold SM (2006). Fatigue in multiple sclerosis: an example of cytokine mediated sickness behaviour?. J Neurol Neurosurg Psychiatry.

[92] Flachenecker P, Bihler I, Weber F, Gottschalk M, Toyka KV, Rieckmann P (2004). Cytokine mRNA expression in patients with multiple sclerosis and fatigue. Mult Scler.

[93] Nagyoszi P, Wilhelm I, Farkas AE, Fazakas C, Dung NT, Haskó J (2010). Expression and regulation of toll-like receptors in cerebral endothelial cells. Neurochem Int.

[94] Andersson A, Covacu R, Sunnemark D, Danilov AI, Dal Bianco A, Khademi M (2008). Pivotal advance: HMGB1 expression in active lesions of human and experimental multiple sclerosis. J Leukoc Biol.

[95] Bsibsi M, Ravid R, Gveric D, van Noort JM (2002). Broad expression of Toll-like receptors in the human central nervous system. J Neuropathol Exp Neurol.

[96] Gironi M, Borgiani B, Mariani E, Cursano C, Mendozzi L, Cavarretta R (2014). Oxidative stress is differentially present in multiple sclerosis courses, early evident, and unrelated to treatment. J Immunol Res.

[97] Miller E, Walczak A, Saluk J, Ponczek MB, Majsterek I (2012). Oxidative modification of patient’s plasma proteins and its role in pathogenesis of multiple sclerosis. Clin Biochem.

[98] Gonsette RE (2008). Neurodegeneration in multiple sclerosis: the role of oxidative stress and excitotoxicity. J Neurol Sci.

[99] Stavropoulou C, Zachaki S, Alexoudi A, Chatzi I, Georgakakos VN, Terzoudi GI (2011). The C609T inborn polymorphism in NAD(P)H:quinone oxidoreductase 1 is associated with susceptibility to multiple sclerosis and affects the risk of development of the primary progressive form of the disease. Free Radic Biol Med.

[100] Fiorini A, Koudriavtseva T, Bucaj E, Coccia R, Foppoli C, Giorgi A (2013). Involvement of oxidative stress in occurrence of relapses in multiple sclerosis: the spectrum of oxidatively modified serum proteins detected by proteomics and redox proteomics analysis. PLoS One.

[101] Oliveira SR, Kallaur AP, Simão AN, Morimoto HK, Lopes J, Panis C (2012). Oxidative stress in multiple sclerosis patients in clinical remission: association with the expanded disability status scale. J Neurol Sci.

[102] Ljubisavljevic S, Stojanovic I, Cvetkovic T, Vojinovic S, Stojanov D, Stojanovic D (2014). Erythrocytes’ antioxidative capacity as a potential marker of oxidative stress intensity in neuroinflammation. J Neurol Sci.

[103] Centonze D, Muzio L, Rossi S, Cavasinni F, De Chiara V, Bergami A (2009). Inflammation triggers synaptic alteration and degeneration in experimental autoimmune encephalomyelitis. J Neurosci.

[104] Lu F, Selak M, O’Connor J, Croul S, Lorenzana C, Butunoi C (2000). Oxidative damage to mitochondrial DNA and activity of mitochondrial enzymes in chronic active lesions of multiple sclerosis. J Neurol Sci.

[105] Mahad D, Lassmann H, Turnbull D (2008). Review: mitochondria and disease progression in multiple sclerosis. Neuropathol Appl Neurobiol.

[106] Reinke S, Broadhurst D, Sykes B, Baker G, Catz I, Warren K (2014). Metabolomic profiling in multiple sclerosis: insights into biomarkers and pathogenesis. Mult Scler.

[107] Lutz NW, Viola A, Malikova I, Confort-Gouny S, Audoin B, Ranjeva JP (2007). Inflammatory multiple-sclerosis plaques generate characteristic metabolic profiles in cerebrospinal fluid. PLoS One.

[108] Lutz NW, Cozzone PJ (2011). Metabolic profiling in multiple sclerosis and other disorders by quantitative analysis of cerebrospinal fluid using nuclear magnetic resonance spectroscopy. Curr Pharm Biotechnol.

[109] Genova H, Rajagopalan V, DeLuca J, Das A, Binder A, Arjunan A (2013). Examination of cognitive fatigue in multiple sclerosis using functional magnetic resonance imaging and diffusion tensor imaging. PLos One.

[110] Kohl AD, Wylie GR, Genova HM, Hillary FG, Deluca J (2009). The neural correlates of cognitive fatigue in traumatic brain injury using functional MRI. Brain Inj.

[111] DeLuca J, Genova H, Capili E, Wylie G (2009). Functional neuroimaging of fatigue. Phys Med Rehabil Clin N Am.

[112] Chaudhuri A, Behan PO (2004). Fatigue in neurological disorders. Lancet.

[113] Messina S, Patti F (2014). Gray matters in multiple sclerosis: cognitive impairment and structural MRI. Mult Scler Int.

[114] Filippi M, Rocca M (2010). MR imaging of gray matter involvement in multiple sclerosis: implications for understanding disease pathophysiology and monitoring treatment efficacy. AJNR Am J Neuroradiol.

[115] Ceccarelli A, Rocca M, Pagani E, Colombo B, Martinelli V, Comi G (2008). A voxel-based morphometry study of grey matter loss in MS patients with different clinical phenotypes. Neuroimage.

[116] Henry R, Shieh M, Okuda D, Evangelista A, Gorno-Tempini M, Pelletier D (2008). Regional grey matter atrophy in clinically isolated syndromes at presentation. J Neurol Neurosurg Psychiatry.

[117] Dalton C, Chard D, Davies G, Miszkiel K, Altmann D, Fernando K (2004). Early development of multiple sclerosis is associated with progressive grey matter atrophy in patients presenting with clinically isolated syndromes. Brain.

[118] Schutzer S, Angel T, Liu T, Schepmoes A, Xie F, Bergquist J (2013). Gray matter is targeted in first-attack multiple sclerosis. PLoS One.

[119] Inglese M, Oesingmann N, Casaccia P, Fleysher L (2011). Progressive multiple sclerosis and gray matter pathology: an MRI perspective. Mt Sinai J Med.

[120] Horakova D, Kalincik T, Dusankova J, Dolezal O (2012). Clinical correlates of grey matter pathology in multiple sclerosis. BMC Neurol.

[121] Debernard L, Melzer T, Van Stockum S, Graham C, Wheeler-Kingshott C, Dalrymple-Alford J (2013). Reduced grey matter perfusion without volume loss in early relapsing-remitting multiple sclerosis. J Neurol Neurosurg Psychiatry.

[122] Calabrese M, Agosta F, Rinaldi F, Mattisi I, Grossi P, Favaretto A (2009). Cortical lesions and atrophy associated with cognitive impairment in relapsing-remitting multiple sclerosis. Arch Neurol.

[123] Damasceno A, Damasceno B, Cendes F (2014). Cerebellar and brain gray-matter damage predicts fatigue in multiple sclerosis (P6. 120). Neurology.

[124] Pellicano C, Gallo A, Li X, Ikonomidou VN, Evangelou IE, Ohayon JM (2010). Relationship of cortical atrophy to fatigue in patients with multiple sclerosis. Arch Neurol.

[125] Inglese M, Park S, Johnson G, Babb J, Miles L, Jaggi H (2007). Deep gray matter perfusion in multiple sclerosis: dynamic susceptibility contrast perfusion magnetic resonance imaging at 3 T. Arch Neurol.

[126] Tedeschi G, Dinacci D, Lavorgna L, Prinster A, Savettieri G, Quattrone A (2007). Correlation between fatigue and brain atrophy and lesion load in multiple sclerosis patients independent of disability. J Neurol Sci.

[127] Roelcke U, Kappos L, Lechner-Scott J, Brunnschweiler H, Huber S, Ammann W (1997). Reduced glucose metabolism in the frontal cortex and basal ganglia of multiple sclerosis patients with fatigue: a 18 F-fluorodeoxyglucose positron emission tomography study. Neurology.

[128] Bakshi R, Miletich RS, Kinkel PR, Emmet ML, Kinkel WR (1998). High-resolution fluorodeoxyglucose positron emission tomography shows both global and regional cerebral hypometabolism in multiple sclerosis. J Neuroimaging.

[129] Blinkenberg M, Rune K, Jensen CV, Ravnborg M, Kyllingsbaek S, Holm S (2000). Cortical cerebral metabolism correlates with MRI lesion load and cognitive dysfunction in MS. Neurology.

[130] Tellez N, Alonso J, Rio J, Tintore M, Nos C, Montalban X (2008). The basal ganglia: a substrate for fatigue in multiple sclerosis. Neuroradiology.

[131] Calabrese M, Rinaldi F, Grossi P, Mattisi I, Bernardi V, Favaretto A (2010). Basal ganglia and frontal/parietal cortical atrophy is associated with fatigue in relapsing–remitting multiple sclerosis. Mult Scler.

[132] Moreno M, Guo F, Ko E, Bannerman P, Soulika A, Pleasure D (2013). Origins and significance of astrogliosis in the multiple sclerosis model, MOG peptide EAE. J Neurol Sci.

[133] Brosnan C (2013). Characteristics of a reactive astrogliosis in multiple sclerosis. Revista Espanola De Esclerosis Multiple.

[134] Hostenbach S, Cambron M, D’haeseleer M, Kooijman R, De Keyser J (2014). Astrocyte loss and astrogliosis in neuroinflammatory disorders. Neurosci Lett.

[135] Oberheim N, Goldman S, Nedergaard M (2012). Heterogeneity of astrocytic form and function. Methods Mol Biol.

[136] Stobart J, Anderson C (2013). Multifunctional role of astrocytes as gatekeepers of neuronal energy supply. Front Cell Neurosci.

[137] Sofroniew M, Vinters H (2010). Astrocytes: biology and pathology. Acta Neuropathol.

[138] Haider L, Simeonidou C, Steinberger G, Hametner S, Grigoriadis N, Deretzi G (2014). Multiple sclerosis deep grey matter: the relation between demyelination, neurodegeneration, inflammation and iron. J Neurol Neurosurg Psychiatry.

[139] Fukuda K, Straus SE, Hickie I, Sharpe MC, Dobbins JG, Komaroff A (1994). The chronic fatigue syndrome: a comprehensive approach to its definition and study. International Chronic Fatigue Syndrome Study Group. Ann Intern Med.

[140] Morris G, Maes M (2013). Case definitions and diagnostic criteria for Myalgic Encephalomyelitis and Chronic fatigue Syndrome: from clinical-consensus to evidence-based case definitions. Neuro Endocrinol Lett.

[141] Holmes GP, Kaplan JE, Gantz NM, Komaroff AL, Schonberger LB, Straus SE (1988). Chronic fatigue syndrome: a working case definition. Ann Intern Med.

[142] Lorusso L, Mikhaylova SV, Capelli E, Ferrari D, Ngonga GK, Ricevuti G (2009). Immunological aspects of chronic fatigue syndrome. Autoimmun Rev.

[143] Klimas NG, Salvato FR, Morgan R, Fletcher MA (1990). Immunologic abnormalities in chronic fatigue syndrome. J Clin Microbiol.

[144] Maes M, Twisk FN, Kubera M, Ringel K (2012). Evidence for inflammation and activation of cell-mediated immunity in myalgic encephalomyelitis/chronic fatigue syndrome (ME/CFS): increased interleukin-1, tumor necrosis factor-α, PMN-elastase, lysozyme and neopteri. J Affect Disord.

[145] Maher KJ, Klimas NG, Fletcher MA (2005). Chronic fatigue syndrome is associated with diminished intracellular perforin. Clin Exp Immunol.

[146] Broderick G, Fuite J, Kreitz A, Vernon SD, Klimas N, Fletcher MA (2010). A formal analysis of cytokine networks in chronic fatigue syndrome. Brain Behav Immun.

[147] Brenu EW, van Driel ML, Staines DR, Ashton KJ, Hardcastle SL, Keane J (2012). Longitudinal investigation of natural killer cells and cytokines in chronic fatigue syndrome/myalgic encephalomyelitis. J Transl Med.

[148] Brenu EW, van Driel ML, Staines DR, Ashton KJ, Ramos SB, Keane J (2011). Immunological abnormalities as potential biomarkers in Chronic Fatigue Syndrome/Myalgic Encephalomyelitis. J Transl Med.

[149] Moss RB, Mercandetti A, Vojdani A (1999). TNF-alpha and chronic fatigue syndrome. J Clin Immunol.

[150] Borish L, Schmaling K, DiClementi JD, Streib J, Negri J, Jones JF (1998). Chronic fatigue syndrome: identification of distinct subgroups on the basis of allergy and psychologic variables. J Allergy Clin Immunol.

[151] Patarca R, Klimas N, Lugtendorf S, Antoni M, Fletcher M (1994). Dysregulated expression of tumor necrosis factor in chronic fatigue syndrome: interrelations with cellular sources and patterns of soluble immune mediator expression. Clin Infect Dis.

[152] Light AR, White AT, Hughen RW, Light KC (2009). Moderate exercise increases expression for sensory, adrenergic, and immune genes in chronic fatigue syndrome patients but not in normal subjects. J Pain.

[153] White AT, Light AR, Hughen RW, Vanhaitsma TA, Light KC (2012). Differences in metabolite-detecting, adrenergic, and immune gene expression after moderate exercise in patients with chronic fatigue syndrome, patients with multiple sclerosis, and healthy controls. Psychosom Med.

[154] Gow JW, Hagan S, Herzyk P, Cannon C, Behan PO, Chaudhuri A (2009). A gene signature for post-infectious chronic fatigue syndrome. BMC Med Genomics.

[155] Maes M, Mihaylova I, Kubera M, Uytterhoeven M, Vrydags N, Bosmans E (2009). Increased 8-hydroxy-deoxyguanosine, a marker of oxidative damage to DNA, in major depression and myalgic encephalomyelitis/chronic fatigue syndrome. Neuro Endocrinol Lett.

[156] Maes M, Mihaylova I, Kubera M, Uytterhoeven M, Vrydags N, Bosmans E (2009). Coenzyme Q10 deficiency in myalgic encephalomyelitis/chronic fatigue syndrome (ME/CFS) is related to fatigue, autonomic and neurocognitive symptoms and is another risk factor explaining the early mortality in ME/CFS due to cardiovascular disorder. Neuro Endocrinol Lett.

[157] Maes M, Kubera M, Uytterhoeven M, Vrydags N, Bosmans E (2011). Increased plasma peroxides as a marker of oxidative stress in myalgic encephalomyelitis/chronic fatigue syndrome (ME/CFS). Med Sci Monit.

[158] Kennedy G, Spence VA, McLaren M, Hill A, Underwood C, Belch JJ (2005). Oxidative stress levels are raised in chronic fatigue syndrome and are associated with clinical symptoms. Free Radic Biol Med.

[159] Shungu DC, Weiduschat N, Murrough JW, Mao X, Pillemer S, Dyke JP (2012). Increased ventricular lactate in chronic fatigue syndrome. III. Relationships to cortical glutathione and clinical symptoms implicate oxidative stress in disorder pathophysiology. NMR Biomed.

[160] Miwa K, Fujita M (2010). Fluctuation of serum vitamin E (alpha-tocopherol) concentrations during exacerbation and remission phases in patients with chronic fatigue syndrome. Heart Vessels.

[161] Fulle S, Pietrangelo T, Mancinelli R, Saggini R, Fanò G (2007). Specific correlations between muscle oxidative stress and chronic fatigue syndrome: a working hypothesis. J Muscle Res Cell Motil.

[162] Myhill S, Booth NE, McLaren-Howard J (2009). Chronic fatigue syndrome and mitochondrial dysfunction. Int J Clin Exp Med.

[163] Behan WM, More IA, Downie I, Gow JW (1995). Mitochondrial studies in the chronic fatigue syndrome. EOS Riv Immunol Immunofarmacol.

[164] McCully KK, Natelson BH (1999). Impaired oxygen delivery to muscle in chronic fatigue syndrome. Clin Sci (Lond).

[165] McCully KK, Natelson BH, Iotti S, Sisto S, Leigh JS (1996). Reduced oxidative muscle metabolism in chronic fatigue syndrome. Muscle Nerve.

[166] Wong R, Lopaschuk G, Zhu G, Walker D, Catellier D, Burton D (1992). Skeletal muscle metabolism in the chronic fatigue syndrome. In vivo assessment by 31P nuclear magnetic resonance spectroscopy. Chest.

[167] Arnold DL, Bore PJ, Radda GK, Styles P, Taylor DJ (1984). Excessive intracellular acidosis of skeletal muscle on exercise in a patient with a post-viral exhaustion/fatigue syndrome. A 31P nuclear magnetic resonance study. Lancet.

[168] Lane RJ, Soteriou BA, Zhang H, Archard LC (2003). Enterovirus related metabolic myopathy: a postviral fatigue syndrome. J Neurol Neurosurg Psychiatry.

[169] Filler K, Lyon D, Bennett J, McCain N, Elswisk R, Lukkahatai N (2014). Association of mitochondrial dysfunction and fatigue: a review of the literature. BBA Clin.

[170] Vermeulen RC, Kurk RM, Visser FC, Sluiter W, Scholte HR (2010). Patients with chronic fatigue syndrome performed worse than controls in a controlled repeated exercise study despite a normal oxidative phosphorylation capacity. J Transl Med.

[171] Vermeulen RC, Vermeulen van Eck IW (2014). Decreased oxygen extraction during cardiopulmonary exercise test in patients with chronic fatigue syndrome. J Transl Med.

[172] Mathew SJ, Mao X, Keegan KA, Levine SM, Smith EL, Heier LA (2009). Ventricular cerebrospinal fluid lactate is increased in chronic fatigue syndrome compared with generalized anxiety disorder: an in vivo 3.0 T (1)H MRS imaging study. NMR Biomed.

[173] Murrough JW, Mao X, Collins KA, Kelly C, Andrade G, Nestadt P (2010). Increased ventricular lactate in chronic fatigue syndrome measured by 1H MRS imaging at 3.0 T. II: comparison with major depressive disorder. NMR Biomed.

[174] Yoshiuchi K, Farkas J, Natelson B (2006). Patients with chronic fatigue syndrome have reduced absolute cortical blood flow. Clin Physiol Funct Imaging.

[175] Machale S, Lawrie S, Cavanagh JT, Glabus MF, Murray CL, Goodwin GM (2000). Cerebral perfusion in chronic fatigue syndrome and depression. Br J Psychiatry.

[176] Ichise M, Salit IE, Abbey SE, Chung DG, Gray B, Kirsh JC (1992). Assessment of regional cerebral perfusion by 99Tcm-HMPAO SPECT in chronic fatigue syndrome. Nucl Med Commun.

[177] de Lange FP, Kalkman JS, Bleijenberg G, Hagoort P, van der Meer JW, Toni I (2005). Gray matter volume reduction in the chronic fatigue syndrome. Neuroimage.

[178] de Lange FP, Koers A, Kalkman JS, Bleijenberg G, Hagoort P, van der Meer JW (2008). Increase in prefrontal cortical volume following cognitive behavioural therapy in patients with chronic fatigue syndrome. Brain.

[179] Okada T, Tanaka M, Kuratsune H, Watanabe Y, Sadato N (2004). Mechanisms underlying fatigue: a voxel-based morphometric study of chronic fatigue syndrome. BMC Neurol.

[180] Kuchinad A, Schweinhardt P, Seminowicz D, Wood P, Chizh B, Bushnell M (2007). Accelerated brain gray matter loss in fibromyalgia patients: premature aging of the brain?. J Neurosci.

[181] Puri BK, Jakeman PM, Agour M, Gunatilake KD, Fernando KA, Gurusinghe AI (2011). Regional grey and white matter volumetric changes in myalgic encephalomyelitis (chronic fatigue syndrome): a voxel-based morphometry 3-T MRI study. Br J Radiol.

[182] Cook DB, O’Connor PJ, Lange G, Steffener J (2007). Functional neuroimaging correlates of mental fatigue induced by cognition among chronic fatigue syndrome patients and controls. Neuroimage.

[183] Caseras X, Mataix-Cols D, Rimes KA, Giampietro V, Brammer M, Zelaya F (2008). The neural correlates of fatigue: an exploratory imaginal fatigue provocation study in chronic fatigue syndrome. Psychol Med.

[184] Siessmeier T, Nix WA, Hardt J, Schreckenberger M, Egle UT, Bartenstein P (2003). Observer independent analysis of cerebral glucose metabolism in patients with chronic fatigue syndrome. J Neurol Neurosurg Psychiatry.

[185] Tirelli U, Chierichetti F, Tavio M, Simonelli C, Bianchin G, Zanco P (1998). Brain positron emission tomography (PET) in chronic fatigue syndrome: preliminary data. Am J Med.

[186] Barnden LR, Crouch B, Kwiatek R, Burnet R, Mernone A, Chryssidis S (2011). A brain MRI study of chronic fatigue syndrome: evidence of brainstem dysfunction and altered homeostasis. NMR Biomed.

[187] Alves G, Wentzel-Larsen T, Larsen JP (2004). Is fatigue an independent and persistent symptom in patients with Parkinson disease?. Neurology.

[188] Pal S, Chaudhuri KR, Trenkwalder C, Forbes A, Bridgman K, DiMarco A (2002). The parkinson’s disease sleep scale (pdss): A new instrument for assessment of sleep, nocturnal disability and daytime sleepiness in parkinson’s disease. Mov Disord.

[189] Friedman JH, Brown RG, Comella C, Garber CE, Krupp LB, Lou JS (2007). Fatigue in Parkinson’s disease: a review. Mov Disord.

[190] van Hilten JJ, Weggeman M, van der Velde EA, Kerkhof GA, van Dijk JG, Roos RA (1993). Sleep, excessive daytime sleepiness and fatigue in Parkinson’s disease. J Neural Transm Park Dis Dement Sect.

[191] Barone P, Antonini A, Colosimo C, Marconi R, Morgante L, Avarello TP (2009). The PRIAMO study: a multicenter assessment of nonmotor symptoms and their impact on quality of life in Parkinson’s disease. Mov Disord.

[192] Hagell P, Brundin L (2009). Towards an understanding of fatigue in Parkinson disease. J Neurol Neurosurg Psychiatry.

[193] Chaudhuri KR, Healy DG, Schapira AH, National Institute for Clinical Excellence (2006). Non-motor symptoms of Parkinson’s disease: diagnosis and management. Lancet Neurol.

[194] Schifitto G, Friedman JH, Oakes D, Shulman L, Comella CL, Marek K (2008). Fatigue in levodopa-naive subjects with Parkinson disease. Neurology.

[195] Dobbs RJ, Charlett A, Purkiss AG, Dobbs SM, Weller C, Peterson DW (1999). Association of circulating TNF-alpha and IL-6 with ageing and parkinsonism. Acta Neurol Scand.

[196] Blum-Degen D, Müller T, Kuhn W, Gerlach M, Przuntek H, Riederer P (1995). Interleukin-1 beta and interleukin-6 are elevated in the cerebrospinal fluid of Alzheimer’s and de novo Parkinson’s disease patients. Neurosci Lett.

[197] Reale M, Iarlori C, Thomas A, Gambi D, Perfetti B, Di Nicola M (2009). Peripheral cytokines profile in Parkinson’s disease. Brain Behav Immun.

[198] Hisanaga K, Asagi M, Itoyama Y, Iwasaki Y (2001). Increase in peripheral CD4 bright + CD8 dull + T cells in Parkinson disease. Arch Neurol.

[199] Bas J, Calopa M, Mestre M, Molleví DG, Cutillas B, Ambrosio S (2001). Lymphocyte populations in Parkinson’s disease and in rat models of parkinsonism. J Neuroimmunol.

[200] Baba Y, Kuroiwa A, Uitti RJ, Wszolek ZK, Yamada T (2005). Alterations of T-lymphocyte populations in Parkinson disease. Parkinsonism Relat Disord.

[201] Scalzo P, Kümmer A, Cardoso F, Teixeira AL (2009). Increased serum levels of soluble tumor necrosis factor-alpha receptor-1 in patients with Parkinson’s disease. J Neuroimmunol.

[202] Dufek M, Hamanová M, Lokaj J, Goldemund D, Rektorová I, Michálková Z (2009). Serum inflammatory biomarkers in Parkinson’s disease. Parkinsonism Relat Disord.

[203] Chen H, O’Reilly EJ, Schwarzschild MA, Ascherio A (2008). Peripheral inflammatory biomarkers and risk of Parkinson’s disease. Am J Epidemiol.

[204] Tansey MG, McCoy MK, Frank-Cannon TC (2007). Neuroinflammatory mechanisms in Parkinson’s disease: potential environmental triggers, pathways, and targets for early therapeutic intervention. Exp Neurol.

[205] Frank-Cannon TC, Alto LT, McAlpine FE, Tansey MG (2009). Does neuroinflammation fan the flame in neurodegenerative diseases?. Mol Neurodegener.

[206] Chung YC, Ko HW, Bok E, Park ES, Huh SH, Nam JH (2010). The role of neuroinflammation on the pathogenesis of Parkinson’s disease. BMB Rep.

[207] Esposito E, Di Matteo V, Benigno A, Pierucci M, Crescimanno G, Di Giovanni G (2007). Non-steroidal anti-inflammatory drugs in Parkinson’s disease. Exp Neurol.

[208] Koprich JB, Reske-Nielsen C, Mithal P, Isacson O (2008). Neuroinflammation mediated by IL-1beta increases susceptibility of dopamine neurons to degeneration in an animal model of Parkinson’s disease. J Neuroinflammation.

[209] Lindqvist D, Kaufman E, Brundin L, Hall S, Surova Y, Hansson O (2012). Non-motor symptoms in patients with Parkinson’s disease - correlations with inflammatory cytokines in serum. PLoS One.

[210] Ferrari CC, Tarelli R (2011). Parkinson’s disease and systemic inflammation. Parkinsons Dis.

[211] Farooqui T, Farooqui AA (2011). Lipid-mediated oxidative stress and inflammation in the pathogenesis of Parkinson’s disease. Parkinsons Dis.

[212] Tsang AH, Chung KK (2009). Oxidative and nitrosative stress in Parkinson’s disease. Biochim Biophys Acta.

[213] Gao HM, Zhou H, Zhang F, Wilson BC, Kam W, Hong JS (2011). HMGB1 acts on microglia Mac1 to mediate chronic neuroinflammation that drives progressive neurodegeneration. J Neurosci.

[214] Ko EA, Min HJ, Shin JS (2012). Interaction of High Mobility Group Box-1 (HMGB1) with α-synuclein and its aggregation [abstract]. J Immunol.

[215] Lindersson EK, Højrup P, Gai WP, Locker D, Martin D, Jensen PH (2004). Alpha-synuclein filaments bind the transcriptional regulator HMGB-1. Neuroreport.

[216] Fellner L, Irschick R, Schanda K, Reindl M, Klimaschewski L, Poewe W (2013). Toll-like receptor 4 is required for α-synuclein dependent activation of microglia and astroglia. Glia.

[217] Kim C, Ho DH, Suk JE, You S, Michael S, Kang J (2013). Neuron-released oligomeric α-synuclein is an endogenous agonist of TLR2 for paracrine activation of microglia. Nat Commun.

[218] Schapira AH, Cooper JM, Dexter D, Clark JB, Jenner P, Marsden CD (1990). Mitochondrial complex I deficiency in Parkinson’s disease. J Neurochem.

[219] Mizuno Y, Ohta S, Tanaka M, Takamiya S, Suzuki K, Sato T (1989). Deficiencies in complex I subunits of the respiratory chain in Parkinson’s disease. Biochem Biophys Res Commun.

[220] Bindoff L, Birch-Machin M, Cartlidge N, Parker W, Turnbull D (1991). Respiratory chain abnormalities in skeletal muscle from patients with Parkinson’s disease. J Neurol Sci.

[221] Penn A, Roberts T, Hodder J, Allen P, Zhu G, Martin W (1995). Generalized mitochondrial dysfunction in Parkinson’s disease detected by magnetic resonance spectroscopy of muscle. Neurology.

[222] Blin O, Desnuelle C, Rascol O, Borg M, Paul H, Azulay JP (1994). Mitochondrial respiratory failure in skeletal muscle from patients with Parkinson’s disease and multiple system atrophy. J Neurol Sci.

[223] Haas R, Nasirian F, Nakano K, Ward D, Pay M, Hill R (1995). Low platelet mitochondrial complex I and complex II/III activity in early untreated Parkinson’s disease. Ann Neurol.

[224] Krige D, Carroll M, Cooper J, Marsden C, Schapira A (1992). Platelet mitochondrial function in Parkinson’s disease. The Royal Kings and Queens Parkinson Disease Research Group. Ann Neurol.

[225] Keeney PM, Xie J, Capaldi RA, Bennett JP (2006). Parkinson’s disease brain mitochondrial complex I has oxidatively damaged subunits and is functionally impaired and misassembled. J Neurosci.

[226] Sherer TB, Betarbet R, Testa CM, Seo BB, Richardson JR, Kim JH (2003). Mechanism of toxicity in rotenone models of Parkinson’s disease. J Neurosci.

[227] Rana M, de Coo I, Diaz F, Smeets H, Moraes CT (2000). An out-of-frame cytochrome b gene deletion from a patient with parkinsonism is associated with impaired complex III assembly and an increase in free radical production. Ann Neurol.

[228] Acín-Pérez R, Bayona-Bafaluy MP, Fernández-Silva P, Moreno-Loshuertos R, Pérez-Martos A, Bruno C (2004). Respiratory complex III is required to maintain complex I in mammalian mitochondria. Mol Cell.

[229] Shults CW, Haas RH, Passov D, Beal MF (1997). Coenzyme Q10 levels correlate with the activities of complexes I and II/III in mitochondria from parkinsonian and nonparkinsonian subjects. Ann Neurol.

[230] Niethammer M, Feigin A, Eidelberg D (2012). Functional neuroimaging in Parkinson’s disease. Cold Spring Harb Perspect Med.

[231] Brooks D (2004). Neuroimaging in Parkinson’s disease. NeuroRx.

[232] Shao N, Yang J, Li J, Shang H (2014). Voxelwise meta-analysis of gray matter anomalies in progressive supranuclear palsy and Parkinson’s disease using anatomic likelihood estimation. Front Hum Neurosci.

[233] Xia J, Miu J, Ding H, Wang X, Chen H, Wang J (2013). Changes of brain gray matter structure in Parkinson’s disease patients with dementia. Neural Regen Res.

[234] Rektorova I, Biundo R, Marecek R, Weis L, Aarsland D, Antonini A (2014). Grey matter changes in cognitively impaired Parkinson’s disease patients. PLos One.

[235] Ellfolk U, Joutsa J, Rinne J, Parkkola R, Jokinen P, Karrasch M (2013). Brain volumetric correlates of memory in early Parkinson’s disease. J Parkinsons Dis.

[236] Nagano-Saito A, Washimi Y, Arahata Y, Kachi T, Lerch JP, Evans AC (2005). Cerebral atrophy and its relation to cognitive impairment in Parkinson disease. Neurology.

[237] Lee H, Kwon K, Kim M, Jang J, Suh S, Koh S (2014). Subcortical grey matter changes in untreated, early stage Parkinson’s disease without dementia. Parkinsonism Relat Disord.

[238] Guevara C, Blain C, Stahl D, Lythgoe D, Leigh P, Barker G (2010). Quantitative magnetic resonance spectroscopic imaging in Parkinson’s disease, progressive supranuclear palsy and multiple system atrophy. Eur J Neurol.

[239] Rango M, Bonifati C, Bresolin N (2005). Parkinson’s disease and brain mitochondrial dysfunction: a functional phosphorus magnetic resonance spectroscopy study. J Cereb Blood Flow Metab.

[240] Fukuda M, Mentis M, Ghilardi MF, Dhawan V, Antonini A, Hammerstad J (2001). Functional correlates of pallidal stimulation for Parkinson’s disease. Ann Neurol.

[241] Fukuda M, Mentis MJ, Ma Y, Dhawan V, Antonini A, Lang AE (2001). Networks mediating the clinical effects of pallidal brain stimulation for Parkinson’s disease: a PET study of resting-state glucose metabolism. Brain.

[242] Huang C, Mattis P, Tang C, Perrine K, Carbon M, Eidelberg D (2007). Metabolic brain networks associated with cognitive function in Parkinson’s disease. Neuroimage.

[243] Hosokai Y, Nishio Y, Hirayama K, Takeda A, Ishioka T, Sawada Y (2009). Distinct patterns of regional cerebral glucose metabolism in Parkinson’s disease with and without mild cognitive impairment. Mov Disord.

[244] Mentis MJ, McIntosh AR, Perrine K, Dhawan V, Berlin B, Feigin A (2002). Relationships among the metabolic patterns that correlate with mnemonic, visuospatial, and mood symptoms in Parkinson’s disease. Am J Psychiatry.

[245] Huang C, Mattis P, Perrine K, Brown N, Dhawan V, Eidelberg D (2008). Metabolic abnormalities associated with mild cognitive impairment in Parkinson disease. Neurology.

[246] Borghammer P, Chakravarty M, Jonsdottir K, Sato N, Matsuda H, Ito K (2010). Cortical hypometabolism and hypoperfusion in Parkinson’s disease is extensive: probably even at early disease stages. Brain Struct Funct.

[247] Peppard RF, Martin WR, Clark CM, Carr GD, McGeer PL, Calne DB (1990). Cortical glucose metabolism in Parkinson’s and Alzheimer’s disease. J Neurosci Res.

[248] Yong SW, Yoon JK, An YS, Lee PH (2007). A comparison of cerebral glucose metabolism in Parkinson’s disease, Parkinson’s disease dementia and dementia with Lewy bodies. Eur J Neurol.

[249] Fernández-Seara M, Mengual E, Vidorreta M, Aznarez-Sanado M, Loayza F, Villagra F (2012). Cortical hypoperfusion in Parkinson’s disease assessed using arterial spin labeled perfusion MRI. Neuroimage.

[250] Kamagata K, Motoi Y, Hori M, Suzuki M, Nakanishi A, Shimoji K (2011). Posterior hypoperfusion in Parkinson’s disease with and without dementia measured with arterial spin labeling MRI. J Magn Reson Imaging.

[251] Marin H, Menza MA (2004). Specific treatment of residual fatigue in depressed patients. Psychiatry (Edgmont).

[252] Marin H, Menza MA (2005). The management of fatigue in depressed patients. Essent Psychopharmacol.

[253] Angst J, Gamma A, Gastpar M, Lépine JP, Mendlewicz J, Tylee A (2002). Gender differences in depression. Epidemiological findings from the European DEPRES I and II studies. Eur Arch Psychiatry Clin Neurosci.

[254] Morrow GR, Hickok JT, Roscoe JA, Raubertas RF, Andrews PL, Flynn PJ (2003). Differential effects of paroxetine on fatigue and depression: a randomized, double-blind trial from the University of Rochester Cancer Center Community Clinical Oncology Program. J Clin Oncol.

[255] Hartz AJ, Bentler SE, Brake KA, Kelly MW (2003). The effectiveness of citalopram for idiopathic chronic fatigue. J Clin Psychiatry.

[256] Fava M, Hoog SL, Judge RA, Kopp JB, Nilsson ME, Gonzales JS (2002). Acute efficacy of fluoxetine versus sertraline and paroxetine in major depressive disorder. J Clin Psychopharmacol.

[257] Wearden AJ, Morriss RK, Mullis R, Strickland PL, Pearson DJ, Appleby L (1998). Randomised, double-blind, placebo-controlled treatment trial of fluoxetine and graded exercise for chronic fatigue syndrome. Br J Psychiatry.

[258] Song C, Halbreich U, Han C, Leonard BE, Luo H (2009). Imbalance between pro- and anti-inflammatory cytokines, and between Th1 and Th2 cytokines in depressed patients: the effect of electroacupuncture or fluoxetine treatment. Pharmacopsychiatry.

[259] Leonard B, Maes M (2012). Mechanistic explanations how cell-mediated immune activation, inflammation and oxidative and nitrosative stress pathways and their sequels and concomitants play a role in the pathophysiology of unipolar depression. Neurosci Biobehav Rev.

[260] Maes M (2011). Depression is an inflammatory disease, but cell-mediated immune activation is the key component of depression. Prog Neuropsychopharmacol Biol Psychiatry.

[261] Miller AH (2010). Depression and immunity: a role for T cells?. Brain Behav Immun.

[262] Gárate I, García-Bueno B, Madrigal JL, Bravo L, Berrocoso E, Caso JR (2011). Origin and consequences of brain Toll-like receptor 4 pathway stimulation in an experimental model of depression. J Neuroinflammation.

[263] Pandey GN, Rizavi HS, Ren X, Bhaumik R, Dwivedi Y (2014). Toll-like receptors in the depressed and suicide brain. J Psychiatr Res.

[264] Gardner A, Boles RG (2011). Beyond the serotonin hypothesis: mitochondria, inflammation and neurodegeneration in major depression and affective spectrum disorders. Prog Neuropsychopharmacol Biol Psychiatry.

[265] Hamilton JP, Etkin A, Furman DJ, Lemus MG, Johnson RF, Gotlib IH (2012). Functional neuroimaging of major depressive disorder: a meta-analysis and new integration of base line activation and neural response data. Am J Psychiatry.

[266] Kempton MJ, Salvador Z, Munafò MR, Geddes JR, Simmons A, Frangou S (2011). Structural neuroimaging studies in major depressive disorder. Meta-analysis and comparison with bipolar disorder. Arch Gen Psychiatry.

[267] Arnone D, McKie S, Elliott R, Juhasz G, Thomas E, Downey D (2012). State-dependent changes in hippocampal grey matter in depression. Mol Psychiatry.

[268] Grieve S, Korgaonkar M, Koslow S, Gordon E, Williams L (2013). Widespread reductions in gray matter volume in depression. Neuroimage Clin.

[269] Bora E, Fornito A, Pantelis C, Yücel M (2012). Gray matter abnormalities in major depressive disorder: a meta-analysis of voxel based morphometry studies. J Affect Disord.

[270] Du M, Wu Q, Yue Q, Li J, Liao Y, Kuang W (2012). Voxelwise meta-analysis of gray matter reduction in major depressive disorder. Prog Neuropsychopharmacol Biol Psychiatry.

[271] Peng J, Liu J, Nie B, Li Y, Shan B, Wang G (2011). Cerebral and cerebellar gray matter reduction in first-episode patients with major depressive disorder: a voxel-based morphometry study. Eur J Radiol.

[272] Walther S, Höfle O, Federspiel A, Horn H, Strik W, Muller T (2011). P02-365-Frontotemporal resting state hypoperfusion in patients with major depression-a study using arterial spin labeling. Eur Psychiatry.

[273] Ho T, Wu J, Shin D, Liu T, Tapert S, Yang G (2013). Altered cerebral perfusion in executive, affective, and motor networks during adolescent depression. J Am Acad Child Adolesc Psychiatry.

[274] Terada S, Oshima E, Sato S, Ikeda C, Nagao S, Hayashi S (2014). Depressive symptoms and regional cerebral blood flow in Alzheimer’s disease. Psychiatry Res.

[275] Ota M, Noda T, Sato N, Hattori K, Teraishi T, Hori H (2014). Characteristic distributions of regional cerebral blood flow changes in major depressive disorder patients: a pseudo-continuous arterial spin labeling (pCASL) study. J Affect Disord.

[276] Martinot J, Hardy P, Feline A, Huret J, Mazoyer B, Attar-Levy D (1990). Left prefrontal glucose hypometabolism in the depressed state: a confirmation. Am J Psychiatry.

[277] Hosokawa T, Momose T, Kasai K (2009). Brain glucose metabolism difference between bipolar and unipolar mood disorders in depressed and euthymic states. Prog Neuropsychopharmacol Biol Psychiatry.

[278] Hirono N, Mori E, Ishii K, Ikejiri Y, Imamura T, Shimomura T (1998). Frontal lobe hypometabolism and depression in Alzheimer’s disease. Neurology.

[279] Steiner J, Walter M, Gos T, Guillemin GJ, Bernstein HG, Sarnyai Z (2011). Severe depression is associated with increased microglial quinolinic acid in subregions of the anterior cingulate gyrus: evidence for an immune-modulated glutamatergic neurotransmission?. J Neuroinflammation.

[280] Krupp LB, LaRocca NG, Muir J, Steinberg AD (1990). A study of fatigue in systemic lupus erythematosus. J Rheumatol.

[281] Krupp LB, LaRocca NG, Muir-Nash J, Steinberg AD (1989). The fatigue severity scale. Application to patients with multiple sclerosis and systemic lupus erythematosus. Arch Neurol.

[282] Krupp LB, Larocca NC, Luft BJ, Halpern JJ (1989). Comparison of neurologic and psychologic findings in patients with Lyme disease and chronic fatigue syndrome. Neurology.

[283] Ramsey-Goldman R, Rothrock N (2010). Fatigue in systemic lupus erythematosus and rheumatoid arthritis. PM R.

[284] Da Costa D, Dritsa M, Bernatsky S, Pineau C, Ménard HA, Dasgupta K (2006). Dimensions of fatigue in systemic lupus erythematosus: relationship to disease status and behavioral and psychosocial factors. J Rheumatol.

[285] Fortin PR, Abrahamowicz M, Neville C, du Berger R, Fraenkel L, Clarke AE (1998). Impact of disease activity and cumulative damage on the health of lupus patients. Lupus.

[286] Wang C, Mayo NE, Fortin PR (2001). The relationship between health related quality of life and disease activity and damage in systemic lupus erythematosus. J Rheumatol.

[287] Al Dhanhani AM, Gignac MA, Su J, Fortin PR (2009). Work disability in systemic lupus erythematosus. Arthritis Rheum.

[288] Panopalis P, Yazdany J, Gillis JZ, Julian L, Trupin L, Hersh AO (2008). Health care costs and costs associated with changes in work productivity among persons with systemic lupus erythematosus. Arthritis Rheum.

[289] Campillo B, Fouet P, Bonnet JC, Atlan G (1990). Submaximal oxygen consumption in liver cirrhosis. Evidence of severe functional aerobic impairment. J Hepatol.

[290] Keyser RE, Rus V, Cade WT, Kalappa N, Flores RH, Handwerger BS (2003). Evidence for aerobic insufficiency in women with systemic Lupus erythematosus. Arthritis Rheum.

[291] Zdrenghea D, Giurgea N, Predescu D, Timiş D, Icuşcă G (1994). Exercise testing in patients with valvular diseases. Rom J Intern Med.

[292] Wysenbeek AJ, Leibovici L, Weinberger A, Guedj D (1993). Fatigue in systemic lupus erythematosus. Prevalence and relation to disease expression. Br J Rheumatol.

[293] Zonana‐Nacach A, Roseman JM, McGwin G, Friedman AW, Baethge BA, Reveille JD. Systemic lupus erythematosus in three ethnic groups. VI: Factors associated with fatigue within 5 years of criteria diagnosis. Lupus. 2000; 9:101–109. 10.1191/096120300678828046 10787006

[294] Marian V, Anolik JH (2012). Treatment targets in systemic lupus erythematosus: biology and clinical perspective. Arthritis Res Ther.

[295] Aringer M, Feierl E, Smolen J (2008). Cytokine blockade-a promising therapeutic option in SLE. Z Rheumatol.

[296] Sabry A, Sheashaa H, El-Husseini A, Mahmoud K, Eldahshan K, George S (2006). Proinflammatory cytokines (TNF-alpha and IL-6) in Egyptian patients with SLE: its correlation with disease activity. Cytokine.

[297] Jacob N, Stohl W (2011). Cytokine disturbances in systemic lupus erythematosus. Arthritis Res Ther.

[298] Aringer M, Smolen J (2004). Tumour necrosis factor and other proinflammatory cytokines in systemic lupus erythematosus: a rationale for therapeutic intervention. Lupus.

[299] Keeling DM, Isenberg DA (1993). Haematological manifestations of systemic lupus erythematosus. Blood Rev.

[300] Wang G, Pierangeli SS, Papalardo E, Ansari GA, Khan MF (2010). Markers of oxidative and nitrosative stress in systemic lupus erythematosus: correlation with disease activity. Arthritis Rheum.

[301] Kim WU, Sreih A, Bucala R (2009). Toll-like receptors in systemic lupus erythematosus; prospects for therapeutic intervention. Autoimmun Rev.

[302] Rahman AH, Eisenberg RA (2006). The role of toll-like receptors in systemic lupus erythematosus. Springer Semin Immunopathol.

[303] Tiffin N, Adeyemo A, Okpechi I (2013). A diverse array of genetic factors contribute to the pathogenesis of systemic lupus erythematosus. Orphanet J Rare Dis.

[304] Dhaouadi T, Sfar I, Haouami Y, Abdelmoula L, Turki S, Hassine LB (2013). Polymorphisms of Toll-like receptor-4 and CD14 in systemic lupus erythematosus and rheumatoid arthritis. Biomark Res.

[305] Morris G, Berk M, Galecki P, Maes M (2014). The emerging role of autoimmunity in myalgic encephalomyelitis/chronic fatigue syndrome (ME/cfs). Mol Neurobiol.

[306] Harel L, Sandborg C, Lee T, von Scheven E (2006). Neuropsychiatric manifestations in pediatric systemic lupus erythematosus and association with antiphospholipid antibodies. J Rheumatol.

[307] Jung R, Segall J, Grazioplene R, Qualls C, Sibbitt W, Roldan C (2010). Cortical thickness and subcortical gray matter reductions in neuropsychiatric systemic lupus erythematosus. PLos One.

[308] Birnbaum J, Petri M, Thompson R, Izbudak I, Kerr D (2009). Distinct subtypes of myelitis in systemic lupus erythematosus. Arthritis Rheum.

[309] Appenzeller S, Li L, Costallat L, Cendes F (2007). Neurometabolic changes in normal white matter may predict appearance of hyperintense lesions in systemic lupus erythematosus. Lupus.

[310] Appenzeller S, Vasconcelos Faria A, Li L, Costallat L, Cendes F (2008). Quantitative magnetic resonance imaging analyses and clinical significance of hyperintense white matter lesions in systemic lupus erythematosus patients. Ann Neurol.

[311] Castellino G, Govoni M, Padovan M, Colamussi P, Borrelli M, Trotta F (2005). Proton magnetic resonance spectroscopy may predict future brain lesions in SLE patients: a functional multi-imaging approach and follow up. Ann Rheum Dis.

[312] Harboe E, Greve O, Beyer M, Goransson L, Tjensvoll A, Maroni S (2008). Fatigue is associated with cerebral white matter hyperintensities in patients with systemic lupus erythematosus. J Neurol Neurosurg Psychiatry.

[313] Gono T, Kawaguchi Y, Yamanaka H (2013). Discoveries in the pathophysiology of neuropsychiatric lupus erythematosus: consequences for therapy. BMC Med.

[314] Gono T, Takarada T, Fukumori R, Kawaguchi Y, Kaneko H, Hanaoka M (2011). NR2-reactive antibody decreases cell viability through augmentation of Ca(2+) influx in systemic lupus erythematosus. Arthritis Rheum.

[315] Kowal C, Degiorgio LA, Lee JY, Edgar MA, Huerta PT, Volpe BT (2006). Human lupus autoantibodies against NMDA receptors mediate cognitive impairment. Proc Natl Acad Sci U S A.

[316] DeGiorgio LA, Konstantinov KN, Lee SC, Hardin JA, Volpe BT, Diamond B (2001). A subset of lupus anti-DNA antibodies cross-reacts with the NR2 glutamate receptor in systemic lupus erythematosus. Nat Med.

[317] Barendregt PJ, Visser MR, Smets EM, Tulen JH, van den Meiracker AH, Boomsma F (1998). Fatigue in primary Sjögren’s syndrome. Ann Rheum Dis.

[318] Markusse HM, Oudkerk M, Vroom TM, Breedveld FC (1992). Primary Sjögren’s syndrome: clinical spectrum and mode of presentation based on an analysis of 50 patients selected from a department of rheumatology. Neth J Med.

[319] Ng WF, Bowman SJ (2010). Primary Sjogren’s syndrome: too dry and too tired. Rheumatology (Oxford).

[320] Giles I, Isenberg D (2000). Fatigue in primary Sjögren's syndrome: is there a link with the fibromyalgia syndrome?. Ann Rheum Dis.

[321] Bax HI, Vriesendorp TM, Kallenberg CG, Kalk WW (2002). Fatigue and immune activity in Sjögren’s syndrome. Ann Rheum Dis.

[322] Tensing EK, Solovieva SA, Tervahartiala T, Nordström DC, Laine M, Niissalo S (2001). Fatigue and health profile in sicca syndrome of Sjögren’s and non-Sjögren’s syndrome origin. Clin Exp Rheumatol.

[323] Haldorsen K, Bjelland I, Bolstad AI, Jonsson R, Brun JG (2011). A five-year prospective study of fatigue in primary Sjögren’s syndrome. Arthritis Res Ther.

[324] Norheim K, Harboe E, Goransson L, Omdal R (2012). Interleukin-1 inhibition and fatigue in primary Sjögren’s syndrome–a double blind, randomised clinical trial. PLos One.

[325] Youinou P, Pers J (2011). Disturbance of cytokine networks in Sjögrens syndrome. Arthritis Res Ther.

[326] Szodoray P, Alex P, Brun J, Centola M, Jonsson R (2004). Circulating cytokines in primary Sjögren’s syndrome determined by a multiplex cytokine array system. Scand J Immunol.

[327] Hagiwara E, Pando J, Ishigatsubo Y, Klinman D (1998). Altered frequency of type 1 cytokine secreting cells in the peripheral blood of patients with primary Sjögren’s syndrome. J Rheumatol.

[328] Katsifis G, Rekka S, Moutsopoulos N, Pillemer S, Wahl S (2009). Systemic and local interleukin-17 and linked cytokines associated with Sjögren’s syndrome immunopathogenesis. Am J Pathol.

[329] Low HZ, Witte T (2011). Aspects of innate immunity in Sjögren’s syndrome. Arthritis Res Ther.

[330] Mavragani CP, Crow MK (2010). Activation of the type I interferon pathway in primary Sjogren’s syndrome. J Autoimmun.

[331] Konttinen YT, Fuellen G, Bing Y, Porola P, Stegaev V, Trokovic N (2012). Sex steroids in Sjögren’s syndrome. J Autoimmun.

[332] Bombardieri M, Pitzalis C (2012). Ectopic lymphoid neogenesis and lymphoid chemokines in Sjogren’s syndrome: at the interplay between chronic inflammation, autoimmunity and lymphomagenesis. Curr Pharm Biotechnol.

[333] Wakamatsu TH, Dogru M, Matsumoto Y, Kojima T, Kaido M, Ibrahim OM (2013). Evaluation of lipid oxidative stress status in Sjögren syndrome patients. Invest Ophthalmol Vis Sci.

[334] Mori K, Iijima M, Koike H, Hattori N, Tanaka F, Watanabe H (2005). The wide spectrum of clinical manifestations in Sjögren’s syndrome-associated neuropathy. Brain.

[335] Lafitte C, Amoura Z, Cacoub P, Pradat-Diehl P, Picq C, Salachas F (2001). Neurological complications of primary Sjögren’s syndrome. J Neurol.

[336] Soliotis FC, Mavragani CP, Moutsopoulos HM (2004). Central nervous system involvement in Sjogren’s syndrome. Ann Rheum Dis.

[337] Manthorpe R, Manthorpe T, Sjöberg S (1992). Magnetic resonance imaging of the brain in patients with primary Sjögren’s syndrome. Scand J Rheumatol.

[338] Alexander EL, Beall SS, Gordon B, Selnes OA, Yannakakis GD, Patronas N (1988). Magnetic resonance imaging of cerebral lesions in patients with the Sjögren syndrome. Ann Intern Med.

[339] Massara A, Bonazza S, Castellino G, Caniatti L, Trotta F, Borrelli M (2010). Central nervous system involvement in Sjögren’s syndrome: unusual, but not unremarkable–clinical, serological characteristics and outcomes in a large cohort of Italian patients. Rheumatology.

[340] Segal B, Mueller B, Zhu X, Prosser R, Pogatchnik B, Holker E (2010). Disruption of brain white matter microstructure in primary Sjögren’s syndrome: evidence from diffusion tensor imaging. Rheumatology.

[341] Pierot L, Sauve C, Leger J, Martin N, Koeger A, Wechsler B (1993). Asymptomatic cerebral involvement in Sjögren’s syndrome: MRI findings of 15 cases. Neuroradiology.

[342] Tzarouchi L, Tsifetaki N, Konitsiotis S, Zikou A, Astrakas L, Drosos A (2011). CNS involvement in primary Sjogren Syndrome: assessment of gray and white matter changes with MRI and voxel-based morphometry. AJR Am J Roentgenol.

[343] Lauvsnes M, Beyer M, Appenzeller S, Greve O, Harboe E, Goransson L (2014). Loss of cerebral white matter in primary Sjögren’s syndrome: a controlled volumetric magnetic resonance imaging study. Eur J Neurol.

[344] Repping-Wuts H, van Riel P, van Achterberg T (2009). Fatigue in patients with rheumatoid arthritis: what is known and what is needed. Rheumatology (Oxford).

[345] Repping-Wuts H, Fransen J, van Achterberg T, Bleijenberg G, van Riel P (2007). Persistent severe fatigue in patients with rheumatoid arthritis. J Clin Nurs.

[346] Repping-Wuts H, Uitterhoeve R, van Riel P, van Achterberg T (2008). Fatigue as experienced by patients with rheumatoid arthritis (RA): a qualitative study. Int J Nurs Stud.

[347] Hewlett S, Cockshott Z, Byron M, Kitchen K, Tipler S, Pope D (2005). Patients’ perceptions of fatigue in rheumatoid arthritis: overwhelming, uncontrollable, ignored. Arthritis Rheum.

[348] Pollard LC, Choy EH, Gonzalez J, Khoshaba B, Scott DL (2006). Fatigue in rheumatoid arthritis reflects pain, not disease activity. Rheumatology (Oxford).

[349] Sariyildiz M, Batmaz I, Bozkurt M, Bez Y, Cetincakmak M, Yazmalar L (2014). Sleep quality in rheumatoid arthritis: relationship between the disease severity, depression, functional status and the quality of life. J Clin Med Res.

[350] Turan Y, Kocaağa Z, Koçyiğit H, Gürgan A, Bayram KB, İpek S (2010). Correlation of fatigue with clinical parameters and quality of life in rheumatoid arthritis. Arch Rheumatol.

[351] Tukaj S, Kotlarz A, Jozwik A, Smolenska Z, Bryl E, Witkowski J (2010). Cytokines of the Th1 and Th2 type in sera of rheumatoid arthritis patients; correlations with anti-Hsp40 immune response and diagnostic markers. Acta Biochim Pol.

[352] Alex P, Szodoray P, Knowlton N, Dozmorov I, Turner M, Frank M (2007). Multiplex serum cytokine monitoring as a prognostic tool in rheumatoid arthritis. Clin Exp Rheumatol.

[353] Chen D, Chen Y, Chen H, Hsieh C, Lin C, Lan J (2011). Increasing levels of circulating Th17 cells and interleukin-17 in rheumatoid arthritis patients with an inadequate response to anti-TNF-alpha therapy. Arthritis Res Ther.

[354] Feldmann M, Maini SR (2008). Role of cytokines in rheumatoid arthritis: an education in pathophysiology and therapeutics. Immunol Rev.

[355] Kremer JM, Westhovens R, Leon M, Di Giorgio E, Alten R, Steinfeld S (2003). Treatment of rheumatoid arthritis by selective inhibition of T-cell activation with fusion protein CTLA4Ig. N Engl J Med.

[356] Liepe K (2012). Efficacy of radiosynovectomy in rheumatoid arthritis. Rheumatol Int.

[357] Moreland LW, Genovese MC, Sato R, Singh A (2006). Effect of etanercept on fatigue in patients with recent or established rheumatoid arthritis. Arthritis Rheum.

[358] Weinblatt ME, Keystone EC, Furst DE, Moreland LW, Weisman MH, Birbara CA (2003). Adalimumab, a fully human anti-tumor necrosis factor alpha monoclonal antibody, for the treatment of rheumatoid arthritis in patients taking concomitant methotrexate: the ARMADA trial. Arthritis Rheum.

[359] Goh F, Midwood K (2011). Intrinsic danger: activation of Toll-like receptors in rheumatoid arthritis. Rheumatology.

[360] Huang Q, Pope R (2009). The role of toll-like receptors in rheumatoid arthritis. Curr Rheumatol Rep.

[361] Brentano F, Kyburz D, Gay S (2009). Toll-like receptors and rheumatoid arthritis. Methods Mol Biol.

[362] Szabo-Taylor K, Nagy G, Eggleton P, Winyard P. Oxidative stress in rheumatoid arthritis. In: Studies on Arthritis and Joint Disorders. Springer Science+Business Media; 2013. p. 145–67 [Alcaraz MJ (Series Editor): Oxidative Stress in Applied Basic Research and Clinical Practice].

[363] Kundu S, Ghosh P, Datta S, Ghosh A, Chattopadhyay S, Chatterjee M (2012). Oxidative stress as a potential biomarker for determining disease activity in patients with rheumatoid arthritis. Free Radic Res.

[364] Hassan S, Gheita T, Kenawy S, Fahim A, El-Sorougy I, Abdou M (2011). Oxidative stress in systemic lupus erythematosus and rheumatoid arthritis patients: relationship to disease manifestations and activity. Int J Rheum Dis.

[365] Tak P, Zvaifler N, Green D, Firestein G (2000). Rheumatoid arthritis and p53: how oxidative stress might alter the course of inflammatory diseases. Immunol Today.

[366] Da Sylva T, Connor A, Mburu Y, Keystone E, Wu G (2005). Somatic mutations in the mitochondria of rheumatoid arthritis synoviocytes. Arthritis Res Ther.

[367] Valcárcel-Ares M, Vaamonde-Garcia C, Riveiro-Naveira R, Lema B, Blanco F, Lopez-Armada M (2010). A novel role for mitochondrial dysfunction in the inflammatory response of rheumatoid arthritis [abstract]. Ann Rheum Dis.

[368] Cillero-Pastor B, Rego-Perez I, Oreiro N, Fernandez-Lopez C, Blanco F (2013). Mitochondrial respiratory chain dysfunction modulates metalloproteases-1,-3 and-13 in human normal chondrocytes in culture. BMC Musculoskelet Disord.

[369] Wartolowska K, Hough M, Jenkinson M, Andersson J, Wordsworth B, Tracey I (2012). Structural changes of the brain in rheumatoid arthritis. Arthritis Rheum.

[370] Bekkelund SI, Pierre-Jerome C, Husby G, Mellgren SI (1995). Quantitative cerebral MR in rheumatoid arthritis. AJNR Am J Neuroradiol.

[371] Mok CC, Lau CS (2003). Pathogenesis of systemic lupus erythematosus. J Clin Pathol.

[372] Voulgarelis M, Tzioufas AG (2010). Current aspects of pathogenesis in Sjögren’s syndrome. Ther Adv Musculoskelet Dis.

[373] Westerlind H, Boström I, Stawiarz L, Landtblom AM, Almqvist C, Hillert J (2014). New data identify an increasing sex ratio of multiple sclerosis in Sweden. Mult Scler.

[374] Bakken I, Tveito K, Gunnes N, Ghaderi S, Stoltenberg C, Trogstad L (2014). Two age peaks in the incidence of chronic fatigue syndrome/myalgic encephalomyelitis: a population-based registry study from Norway 2008-2012. BMC Med.

[375] Piccinelli M, Wilkinson G (2000). Gender differences in depression. Critical review. Br J Psychiatry.

[376] Lubomski M, Louise Rushworth R, Lee W, Bertram KL, Williams DR (2014). Sex differences in Parkinson’s disease. J Clin Neurosci.

[377] Benkler M, Agmon-Levin N, Hassin-Baer S, Cohen OS, Ortega-Hernandez OD, Levy A (2012). Immunology, autoimmunity, and autoantibodies in Parkinson’s disease. Clin Rev Allergy Immunol.

[378] Oertelt-Prigione S (2012). The influence of sex and gender on the immune response. Autoimmun Rev.

[379] Munoz-Cruz S, Togno-Pierce C, Morales-Montor J (2011). Non-reproductive effects of sex steroids: their immunoregulatory role. Curr Top Med Chem.

[380] Berghella A, Contasta I, Del Beato T, Pellegrini P (2012). The discovery of how gender influences age immunological mechanisms in health and disease, and the identification of ageing gender-specific biomarkers, could lead to specifically tailored treatment and ultimately improve therapeutic success rates. Immun Ageing.

[381] Sárvári M, Hrabovszky E, Kalló I, Solymosi N, Tóth K, Likó I (2011). Estrogens regulate neuroinflammatory genes via estrogen receptors α and β in the frontal cortex of middle-aged female rats. J Neuroinflammation.

[382] Schiebinger L, Schraudner M (2011). Interdisciplinary approaches to achieving gendered innovations in science, medicine, and engineering. Interdisc Science Rev.

[383] Pittman P (2002). Aluminum-containing vaccine associated adverse events: role of route of administration and gender. Vaccine.

[384] Reif D, Motsinger-Reif A, McKinney B, Rock M, Crowe J, Moore J (2008). Integrated analysis of genetic and proteomic data identifies biomarkers associated with adverse events following smallpox vaccination. Genes Immun.

[385] Vera-Lastra O, Medina G, Cruz-Dominguez M, Jara L, Shoenfeld Y (2013). Autoimmune/inflammatory syndrome induced by adjuvants (Shoenfeld’s syndrome): clinical and immunological spectrum. Expert Rev Clin Immunol.

[386] Nancy A, Shoenfeld Y (2008). Chronic fatigue syndrome with autoantibodies–the result of an augmented adjuvant effect of hepatitis-B vaccine and silicone implant. Autoimmun Rev.

[387] Kool M, Petrilli V, De Smedt T, Rolaz A, Hammad H, van Nimwegen M (2008). Cutting edge: alum adjuvant stimulates inflammatory dendritic cells through activation of the NALP3 inflammasome. J Immunol.

[388] Yan Z, Zhang Q, Xu L, Wu W, Ren W, Liu LH (2010). Involvement of Toll-like receptor in silica-induced tumor necrosis factor alpha release from human macrophage cell line. Zhonghua Lao Dong Wei Sheng Zhi Ye Bing Za Zhi.

[389] Perricone C, Agmon-Levin N, Shoenfeld Y (2013). Novel pebbles in the mosaic of autoimmunity. BMC Med.

[390] Pfreundschuh M, Muller C, Zeynalova S, Kuhnt E, Wiesen M, Held G (2013). Suboptimal dosing of rituximab in male and female patients with DLBCL. Blood.

[391] Anderson G (2014). Gender differences in pharmacological response. Int Rev Neurobiol.

[392] Sivro A, Lajoie J, Kimani J, Jaoko W, Plummer F, Fowke K (2013). Age and menopause affect the expression of specific cytokines/chemokines in plasma and cervical lavage samples from female sex workers in Nairobi. Kenya. Immun Ageing.

[393] Stasi R (2010). Rituximab in autoimmune hematologic diseases: not just a matter of B cells. Semin Hematol.

[394] Tsuda M, Moritoki Y, Lian Z, Zhang W, Yoshida K, Wakabayashi K (2012). Biochemical and immunologic effects of rituximab in patients with primary biliary cirrhosis and an incomplete response to ursodeoxycholic acid. Hepatology.

[395] van de Veerdonk F, Lauwerys B, Marijnissen R, Timmermans K, Di Padova F, Koenders MI (2011). The anti-CD20 antibody rituximab reduces the Th17 cell response. Arthritis Rheum.

[396] Yamamoto A, Sato K, Miyoshi F, Shindo Y, Yoshida Y, Yokota K (2009). Analysis of cytokine production patterns of peripheral blood mononuclear cells from a rheumatoid arthritis patient successfully treated with rituximab. Mod Rheumatol.

[397] Morris G, Anderson G, Dean O, Berk M, Galecki P, Martin-Subero M (2014). The glutathione system: a new drug target in neuroimmune disorders. Mol Neurobiol.

[398] Greco CM, Nakajima C, Manzi S (2013). Updated review of complementary and alternative medicine treatments for systemic lupus erythematosus. Curr Rheumatol Rep.

[399] Maes M, Fišar Z, Medina M, Scapagnini G, Nowak G, Berk M (2012). New drug targets in depression: inflammatory, cell-mediated immune, oxidative and nitrosative stress, mitochondrial, antioxidant, and neuroprogressive pathways. And new drug candidates–Nrf2 activators and GSK-3 inhibitors. Inflammopharmacology.

[400] Puri BK, Holmes J, Hamilton G (2004). Eicosapentaenoic acid-rich essential fatty acid supplementation in chronic fatigue syndrome associated with symptom remission and structural brain changes. Int J Clin Pract.

[401] Duffy EM, Meenagh GK, McMillan SA, Strain JJ, Hannigan BM, Bell AL (2004). The clinical effect of dietary supplementation with omega-3 fish oils and/or copper in systemic lupus erythematosus. J Rheumatol.

[402] Kremer JM (2000). n-3 fatty acid supplements in rheumatoid arthritis. Am J Clin Nutr.

[403] Lopresti AL, Maes M, Maker GL, Hood SD, Drummond PD (2014). Curcumin for the treatment of major depression: a randomised, double-blind, placebo controlled study. J Affect Disord.

[404] Chandran B, Goel A (2012). A randomized, pilot study to assess the efficacy and safety of curcumin in patients with active rheumatoid arthritis. Phytother Res.

